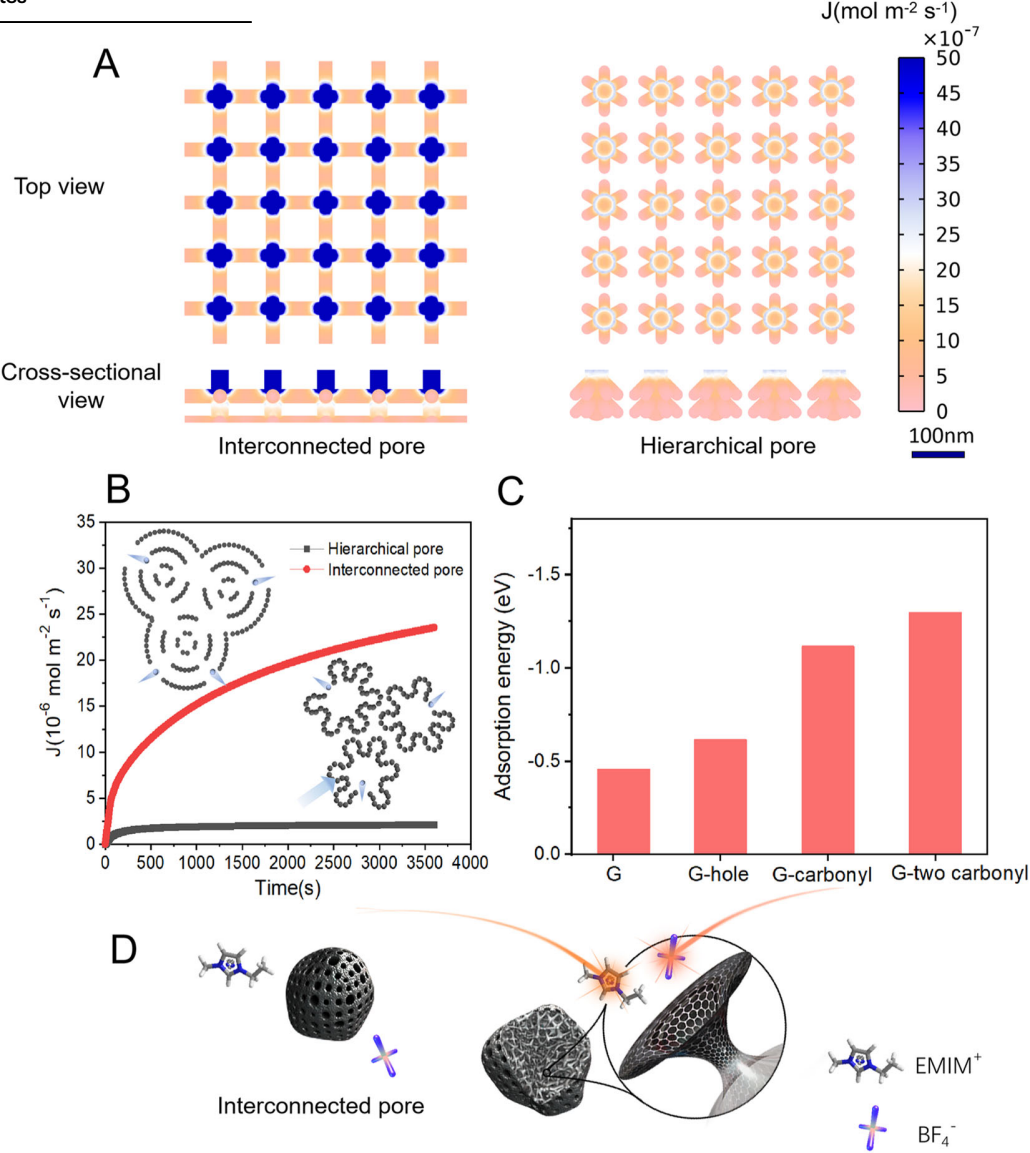# Author Correction: Sub-millisecond lithiothermal synthesis of graphitic meso–microporous carbon

**DOI:** 10.1038/s41467-024-48805-0

**Published:** 2024-05-20

**Authors:** Huimin Zhang, Jingyi Qiu, Jie Pang, Gaoping Cao, Bingsen Zhang, Li Wang, Xiangming He, Xuning Feng, Shizhou Ma, Xinggao Zhang, Hai Ming, Zhuangnan Li, Feng Li, Hao Zhang

**Affiliations:** 1 Beijing Key Laboratory of Advanced Chemical Energy Storage Technologies and Materials, Research Institute of Chemical Defense, Beijing, China; 2 School of Energy Science and Technology, Henan University, Zhengzhou, China; 3 Shenyang National Laboratory for Materials Science, Institute of Metal Research, Chinese Academy of Sciences, Shenyang, China; 4 Institute of Nuclear and New Energy Technology, Tsinghua University, Beijing, China; 5 Department of Material Science and Metallurgy, University of Cambridge, Cambridge, UK

**Keywords:** Materials for energy and catalysis, Energy storage

Correction to: *Nature Communications* 10.1038/s41467-024-47916-y, published online 25 April 2024

In this article, the x-axis for Fig. 4C was missing; the figure should have appeared as shown below. The original article has been corrected.