# Supplementary material for: Sub-millisecond lithiothermal synthesis of graphitic meso–microporous carbon
Source: Nat Commun. 2024 Apr 25;15:3491. doi: 10.1038/s41467-024-47916-y (PMC11045851; doi:10.1038/s41467-024-47916-y)
Supplement: Supplementary file 1 — Supplementary Information [file 41467_2024_47916_MOESM1_ESM.pdf]

Supplementary Information

Sub-millisecond lithiothermal synthesis of graphitic meso-microporous carbon

Huimin Zhang[1], Jingyi Qiu[1], Jie Pang[2], Gaoping Cao[1], Bingsen Zhang[3], Li Wang[4], Xiangming He[4], Xuning Feng[4], Shizhou Ma[1], Xinggao Zhang[1], Hai Ming[1], Zhuangnan Li[5], Feng Li[3*], Hao Zhang[1*]

[1] Beijing Key Laboratory of Advanced Chemical Energy Storage Technologies and Materials, Research Institute of Chemical Defense; Beijing, 100191, China.

[2] School of Energy Science and Technology, Henan University, Zhengzhou, 450046, China.

[3] Shenyang National Laboratory for Materials Science, Institute of Metal Research, Chinese Academy of Sciences; Shenyang, 110016, China.

[4] Institute of Nuclear and New Energy Technology, Tsinghua University; Beijing, 100084, China.

[5] Department of Material Science and Metallurgy, University of Cambridge; Cambridge CB3 0FS, UK.

*Corresponding author. Email: dr.h.zhang@hotmail.com; fli@imr.ac.cn.

Table of Contents

Supplementary Methods…… 3
Supplementary Figure 1…… 5
Supplementary Figure 2…… 6
Supplementary Figure 3…… 7
Supplementary Figure 4…… 8
Supplementary Note 1…… 9
Supplementary Figure 5…… 10
Supplementary Figure 6…… 11
Supplementary Figure 7…… 12
Supplementary Figure 8…… 13
Supplementary Figure 9…… 14
Supplementary Figure 10…… 15
Supplementary Figure 11…… 16
Supplementary Figure 12…… 17
Supplementary Figure 13…… 18
Supplementary Figure 14…… 19
Supplementary Figure 15…… 20
Supplementary Figure 16…… 21
Supplementary Figure 17…… 22
Supplementary Figure 18…… 23
Supplementary Figure 19…… 24
Supplementary Figure 20…… 25
Supplementary Figure 21…… 26
Supplementary Figure 22…… 27
Supplementary Figure 23…… 28
Supplementary Figure 24…… 29
Supplementary Figure 25…… 30
Supplementary Figure 26…… 31
Supplementary Figure 27…… 32
Supplementary Figure 28…… 33
Supplementary Figure 29…… 34
Supplementary Figure 30…… 35
Supplementary Figure 31…… 36
Supplementary Figure 32…… 37
Supplementary Figure 33…… 38
Supplementary Figure 34…… 39
Supplementary Figure 35…… 40
Supplementary Figure 36…… 41
Supplementary Table 1…… 42
Supplementary Table 2…… 43
Supplementary Table 3…… 44
Supplementary References…… 46

Supplementary Methods

Simulations of electrolyte ion distribution

The geometry models are built by the COMSOL Multiphysics 5.5 software to simulate the mass transfer of electrolyte in different pore morphologies, in which the electrolyte layer is set to 20 mm, and the electrode layer is set to 5 mm. For this model, the setup of the electrode domain is critical, because both electrode reactions and channel flow, which have significant effect on electrolyte transport, occur here. Another important part is to establish the correct channel form close to the actual situation. The key setting of the simulation is listed as follows.

**Material properties**	
Applied Current	$1*10^{-3}$ A
Electrolyte conductivity	0.35 S/m
Cell temperature	293.15 K
Reference concentration	1 mol/L
Initial ion concentration, c_ion	$1*10^{-6}$ mol/L
Initial electrode equilibrium potential	-0.76[V]+R_const*T/(2*F_const)*log(c_ion/c_ref)
Cathodic Transfer Coefficient	1.5
Exchange current density on electrode	10 A/m^2
electrode reference equilibrium potential	-0.76 V
electrode reference exchange current density	10 A/m^2

The flow field distribution of the electrolyte was modeled using the COMSOL Multiphysics finite-element-based solver (http://cn.comsol.com). The simulation of the mass transfer of electrolytes in porous electrodes was utilized secondary current module and dilute substance transfer module. This model needs to consider both mass transfer caused by diffusion, convection, and migration. Therefore, the equations to be solved and the equations governed by the variables can be given by

$$\nabla \cdot N_i = 0$$
$$-\mathrm{D}_i \nabla \mathrm{c}_i - \mathrm{z}_i \mathrm{m}_i \mathrm{F} \mathrm{c}_i \nabla \varphi_l + \mathrm{c}_i \mathbf{u} = \mathrm{N}_i \tag{1}$$

where c_i represents the concentration of ion i (SI unit: mol/m^3), z_i valence, D_i its diffusivity (SI unit: m^2/s), m_i mobility (SI unit: mol·m^2(s·V·A)), F denotes the Faraday constant (SI unit: As/mol), φ_l the ionic potential, and $\boldsymbol{u}$ the velocity vector (SI unit: m/s). The three terms in the above equation are usually represents three transfer mechanisms: diffusion, migration, and convection.

In order to introduce mass transfer dependence in the model, it is presumed that the oxidized substance on the anode is limited by mass transfer, and its local concentration c (SI unit: mol / m^3) affects the electrode kinetics. Therefore, the Butler-Volmer expression on the anode is related to concentration, and the expression now becomes:

$$i_a = i_0 \left(\frac{\mathrm{c}}{c_0} \exp \frac{(\,\eta(1-\beta)\mathrm{F})}{\mathrm{R}T} \right) - \exp\left(\frac{\eta\beta\mathrm{F}}{\mathrm{R}T}\right) \tag{2}$$

Herein, T is temperature and R is gas constant (SI unit: J/(K·mol). i_0, the exchange current density, (SI unit: A/m^2), and β, the symmetry factor, are reaction and electrode dependent and therefore are different for each electrode. c_0 (SI unit: mol/m^3) denotes reference concentration.

Overpotential η is the difference between the electrode potential and the equilibrium potential of the electrode reaction and is defined as:

$$\eta = E_{electrode} - E_{eq} \tag{3}$$

This model also introduces momentum conservation equation to describe convection. In this case, assuming a steady state incompressible laminar flow, the Navier-Stokes equation is used:

$$-\nabla \cdot \mu(\nabla\mathbf{u} + (\nabla\mathbf{u})^T) + \rho(\boldsymbol{u} \cdot \nabla)\mathbf{u} + \nabla p = 0 \tag{4}$$

where μ is the dynamic viscosity (SI unit: Ns/m^2), ρ density (SI unit: kg/m^3) and p pressure (SI unit: Pa).

Simulations for temperature field of lithiothermal reaction

This geometry models are developed in the two modules, non-isothermal flow and heat transfer in the COMSOL Multiphysics 5.5 software to simulate the explosive reaction, which are two-dimensional axisymmetric model. In this model, three domains are set, including the air domain, the reaction domain and the heating domain, which are 1 dm, 0.5 dm and 0.3 dm in length and 0.5 dm, 0.05 dm and 0.02 dm in height, respectively.

Heat transfer in three domains is controlled by heat conduction:

$$\rho A \frac{\partial T}{\partial t} = \nabla \cdot k \nabla T \tag{5}$$

where c_i, kg m^{-3} and k, W m^{-1} K^{-1} represents the density and heat conductivity of heat-transfer material, respectively; A represents its specific heat capacity.

For this model, the detail process of chemical reaction is ignored because this simulation aims to analyze the temperature field distribution and velocity field distribution, in which the chemical process can be simplified to heat source that changes with the time. A piecewise function is utilized to indicate the heat source, including two intersecting linear functions and zero functions at both ends are composed, in which the highest peak value of the intersection that is defined using the reaction enthalpy change refers to the result of simulation calculation. This reaction domain was set to contain 1 mol of reactant mixture, the heat source function is constructed as

$$\mathrm{Q} = \mathrm{f}(t) = \frac{\left[\frac{t_R}{2}\left|\frac{t_R}{2}\left(t - \frac{z}{v_{SHS}}\right)\right|\right]E}{\left({}^{t_R}/_{2}\right)^2} \qquad \left(0 \le t - \frac{z}{v_{SHS}} \le t_R\right) \tag{6}$$

in which v_{SHS}, cm s^{-1} is propagating rate for combustion wave, t_R is reaction time for exothermic peak, and E is heat release, kJ m^{-3}.

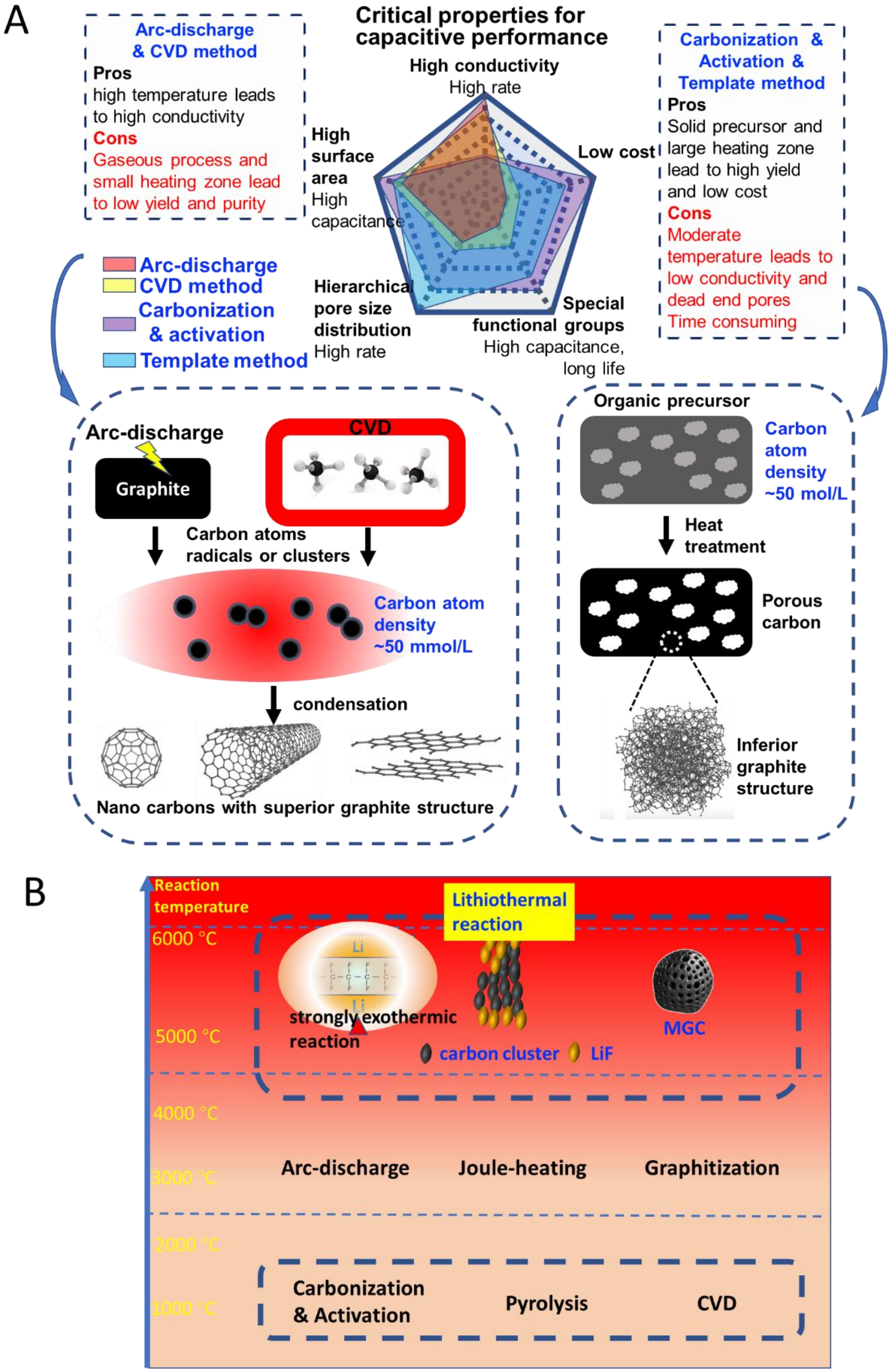

Supplementary Fig. 1. (A) Advantages and limits of various methodologies for capacitive carbon preparation. **(B)** Reaction temperature for various methodologies (*1*).

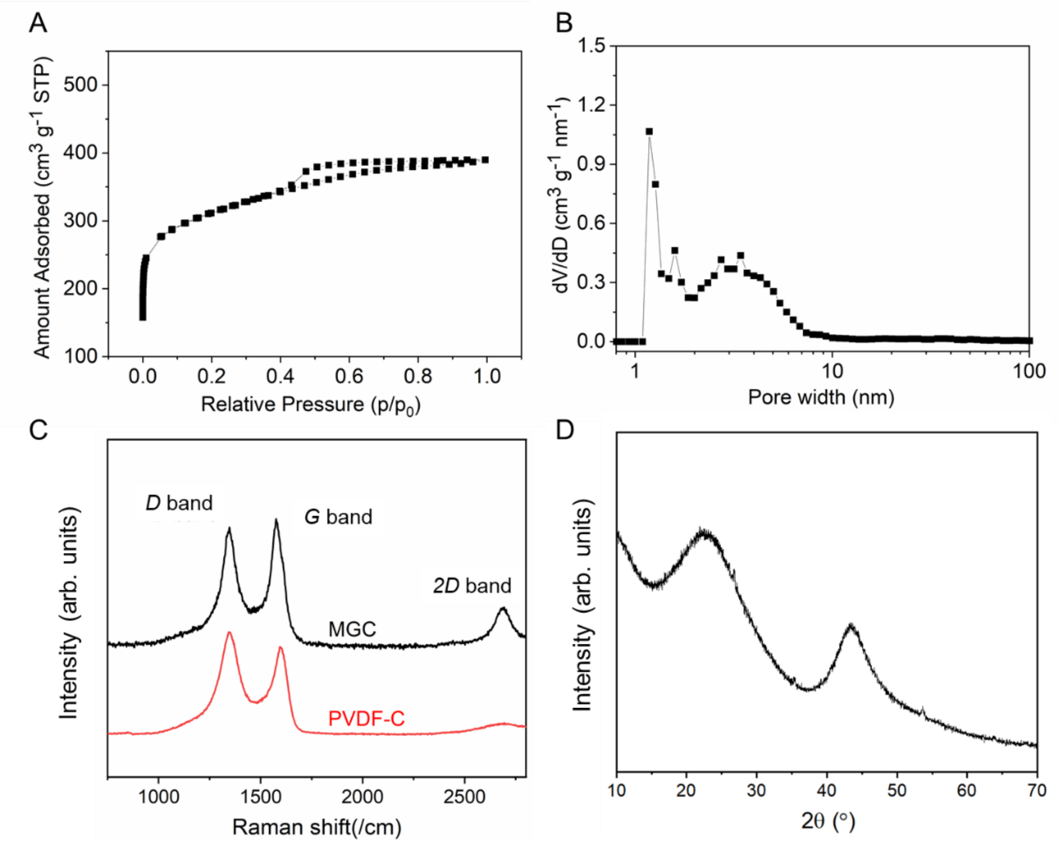

Supplementary Fig. 2. Characterization of porous carbon prepared by carbonizing PVDF precursor at 1000 ˚C in Ar (PVDF-C). (A) N_2 adsorption/desorption isotherm and **(B)** pore size distribution of PVDF-C. **(C)** XRD pattern and **(D)** Raman spectra from PVDF-C. Direct pyrolysis of PVDF tends to yield low graphitized porous carbonaceous products under continuous heating because of its alternating CF_2 and CH_2 groups (*2*).

PVDF-C exhibited a typical type-IV isotherm with an obvious type-H4 hysteresis loop in the relative pressure range of 0.45-1.0, and displayed micro-meso pore size distribution of 1-10nm (Supplementary Fig. 2A, B). The Raman spectra (Supplementary Fig. 2C) exhibited two broad bands of the *D*-band peak and *G* band peak, and there exists no obvious 2*D* band. XRD patterns (Supplementary Fig. 2D) showed the broad peaks at 24° and 43°, which confirm their amorphous structure.

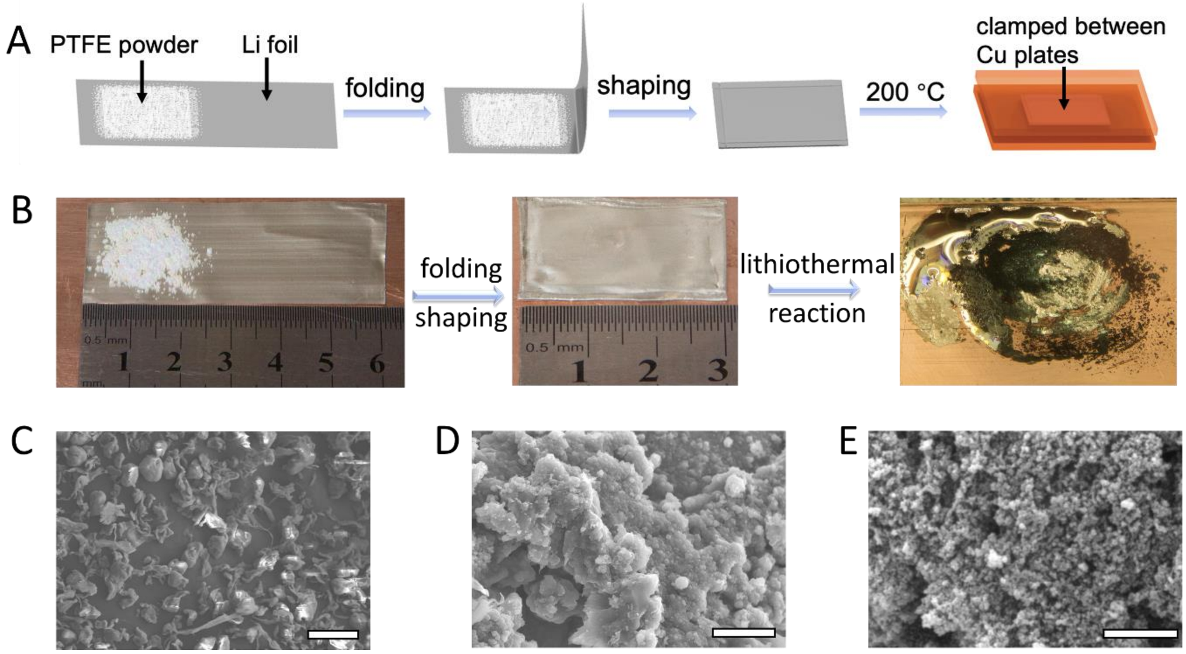

Supplementary Fig. 3. Lithiothermal preparation process. (A) Schematic presents folding PTFE powder by Li foil, shaping the reactant envelop, and clamping it by two Cu plates with 1 MPa. When the Cu plates are heated to 200 °C with a lateral shear, lithium foil melts, and then a fierce lithiothermal reaction will happen in this semi-closed system. **(B)** Optical pictures of Li wrapper/PTFE filling envelop and the products after lithiothermal reaction, which is composed of LiF, carbon, and excessive Li metal. SEM images of **(C)** PTFE powder, **(D)**, **(E)** products after lithiothermal reaction. Scale bar: 20 μm (C), 5 μm (D) and 500 nm (E).

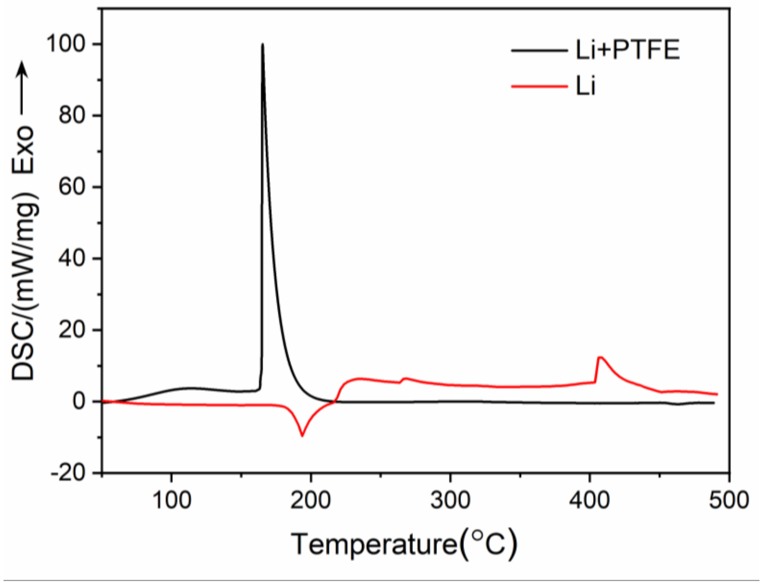

Supplementary Fig. 4. DSC curves of Li/PTFE and Li under pressure in argon atmosphere. The major exothermic peak at 170 °C of Li/PTFE is much larger than the melting peak at 195 °C of Li metal, which indicates that Li and PTFE have high reactivity, large reaction heat and fast mass rate. It is calculated that the adiabatic temperature for the lithiothermal reaction is higher than 3000K.

Supplementary Note 1

Theoretical calculation of adiabatic temperature (T_{ad}) helps to understand the reaction process between PTFE and Li metal. For an adiabatic reaction, T_{ad} can be readily estimated from:

$$-\Delta_r H_{298}^{\theta} = \sum n_i \left(H_T^{\theta} - H_{298}^{\theta}\right)_{\text{product } i} \quad (7)$$

where ΔH_{298}^{θ} is standard molar reaction enthalpy for the reaction, n_i is the molar coefficient of product i, $H_T^{\theta} - H_{298}^{\theta}$ is molar enthalpy of formation at temperature T.

$$H_T^{\theta} - H_{298}^{\theta} = \int_{298}^{T_m} C_p dT + \Delta H_m + \int_{T_m}^{T_b} C_p' dT + \Delta H_b + \int_{T_b}^{T_{ad}} C_p'' dT \quad (8)$$

Where ΔH_m is molar enthalpy of fusion, ΔH_b is molar enthalpy of evaporation, T_m is melting point, T_b is boiling point, C_p, C_p', C_p'' is the molar specific heat capacity of products at different states.

Specially, for the reaction as follow:

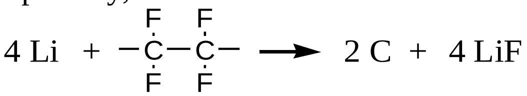

Where $\Delta_r H_{298}^{\theta} = 4\,\Delta_f H_{298}^{\theta}(\text{LiF}) - \Delta_f H_{298}^{\theta}(\text{C}_2\text{F}_4)$, $\Delta_f H_{298}^{\theta}$(LiF)=-616.931 kJ mol^{-1}, $\Delta_f H_{298}^{\theta}(\text{C}_2\text{F}_4)$=-658.562 kJ mol^{-1}, ΔH_m(LiF)= 27.087 kJ mol^{-1}, $\Delta H_m(\text{C}_2\text{F}_4)$= 213.41 kJ mol^{-1}, T_m(LiF)=1121.15 K, T_b(LiF)=1954.15 K.

The molar specific heat capacity of products in reaction above.

	C_p(J mol^{-1} K^{-1})	C_p'(J mol^{-1} K^{-1})	C_p''(J mol^{-1} K^{-1})
LiF	51.859	64.183	38.4155
C	15.352	23.301	25.1025

After calculation, $\boldsymbol{T_{ad}}$**=3909 K.**

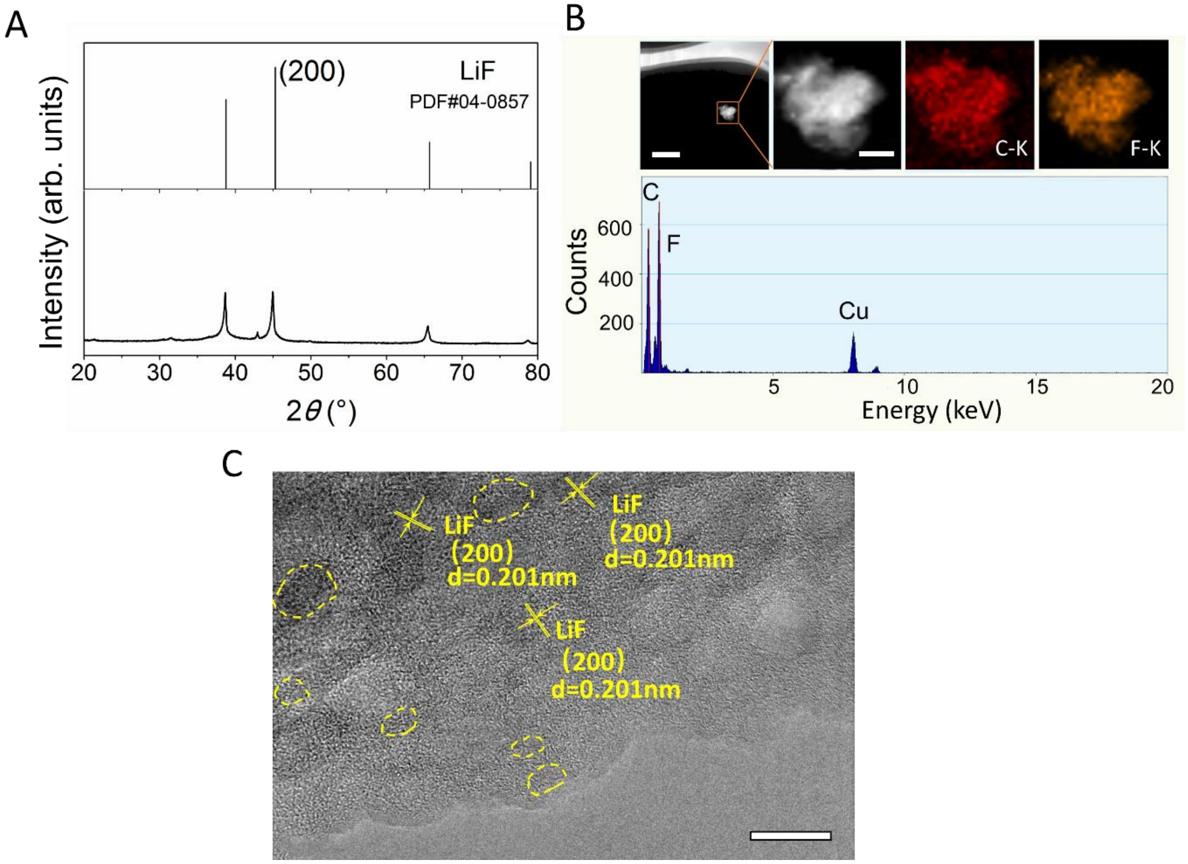

Supplementary Fig. 5. Characterization of lithiothermal reaction products in Ar. (A) XRD pattern indicates the existence of LiF. **(B)** EDS mapping of a product particle, demonstrating that the product is rich in F and C Elements. **(C)** TEM image of the product. Tremendous LiF nanoparticles with diameter of 3~5 nm are observed. Scale bar: 500 nm and 100nm(enlarged view) (B) and 5 nm (C).

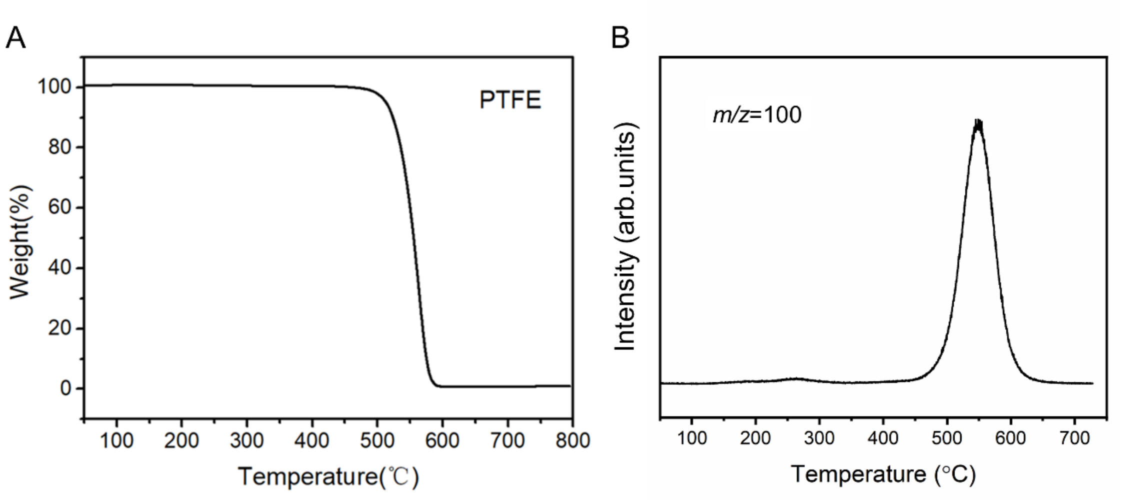

Supplementary Fig. 6. (A) TG curve of PTFE under N_2 atmosphere. Around 100 wt.% mass loss around 550 ˚C corresponds to complete pyrolysis of PTFE. **(B)** The TG-mass spectra of PTFE. During pyrolysis process, the gas decomposed from PTFE was mainly C_2F_4.

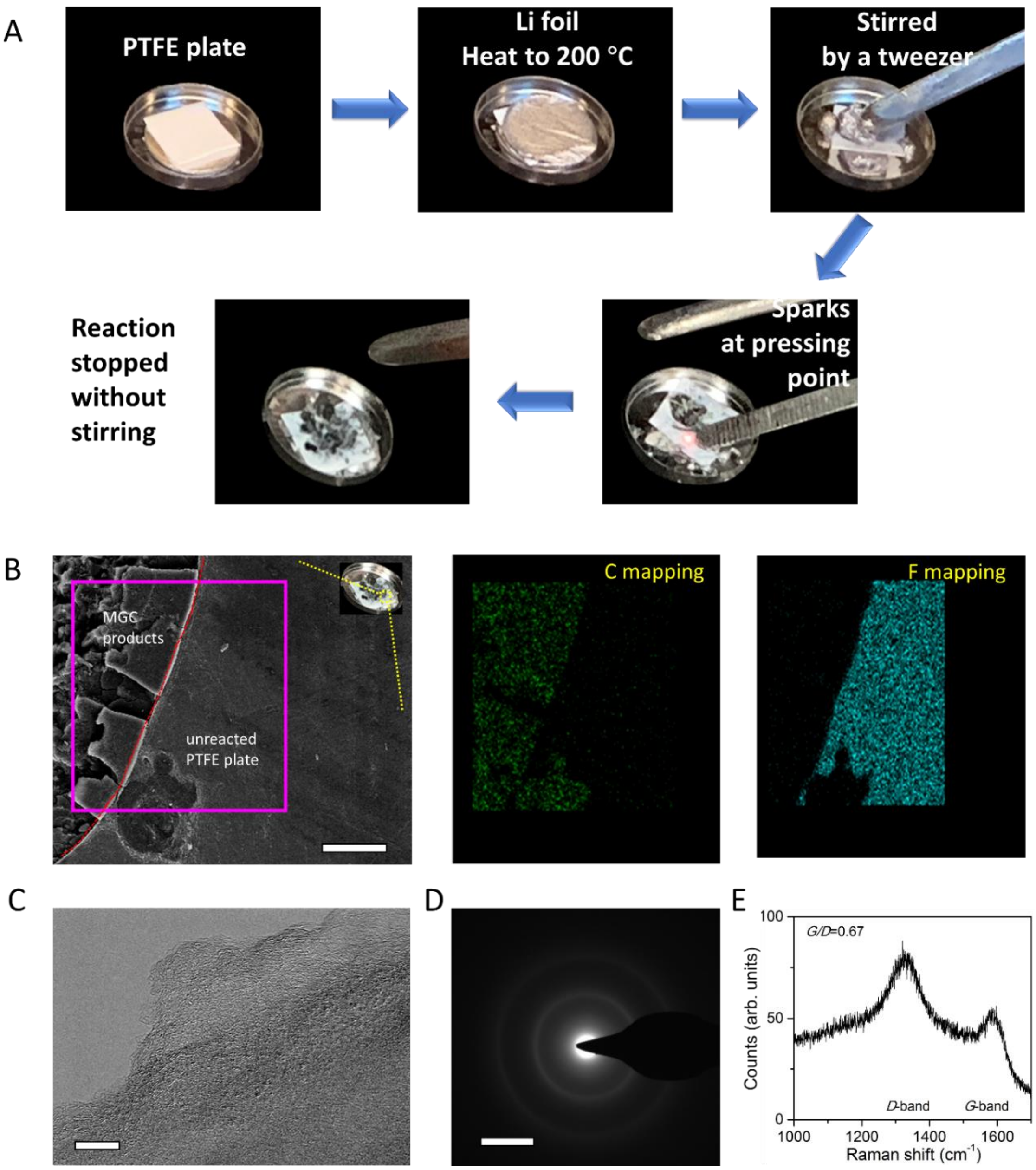

Supplementary Fig. 7. (A) Lithiothermal reaction between PTFE plate with Li foil. It is obvious that PTFE plate shows very low reactivity. There is no reaction between PTFE plate and molten Li foil without external force. Sparks are observed when use a Tweezer to press the molten Li against PTFE plate. However, reaction stops without propagating as soon as taking the tweezer away. This result demonstrates that the high reaction interface between PTFE and molten Li, which is attributed to the high surface area of PTFE powder, is critical for the self-propagating of the reaction. **(B)** SEM and related C and F EDS images of the product on PTFE plate after reaction, the inset presents an optical image of the PTFE plate after reaction. **(C)** TEM image, **(D)** corresponding selected-area electron diffraction pattern, and **(E)** Raman spectra of carbon product. scale bar: 20 µm (B), 10 nm (C), 5 1/nm (D).

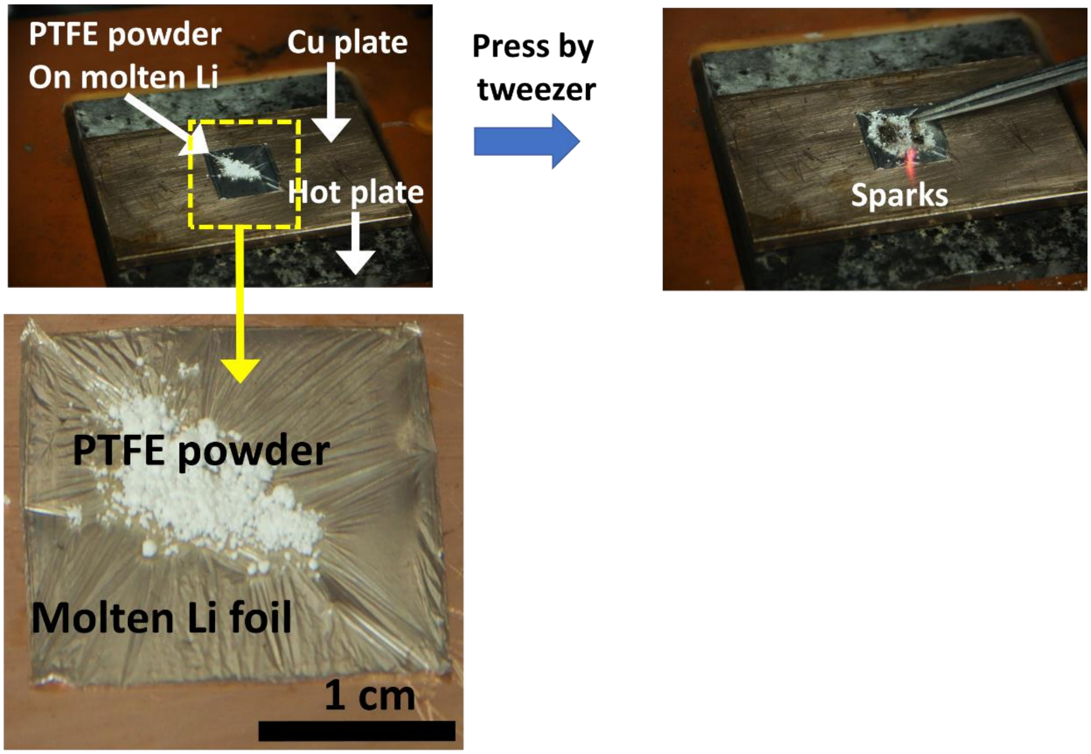

Supplementary Fig. 8. Optical pictures show lithiothermal reaction between molten Li and PTFE powder ignited by the pressing of tweezer. In a glove box, 0.1 g PTFE powder was loaded onto a 2 cm × 2 cm Li foil, which was placed on the center of a Cu plate. The Cu plate was put onto a hot plate and heated to 200 °C. It should be noted that PTFE powder did not react with molten Li without external interference. Sparks are observed when using a tweezer to impinge the molten Li against PTFE plate. This result demonstrates that an external trigger can ensure the high reaction interface between PTFE powder and molten Li, and then sustain the self-propagating reaction.

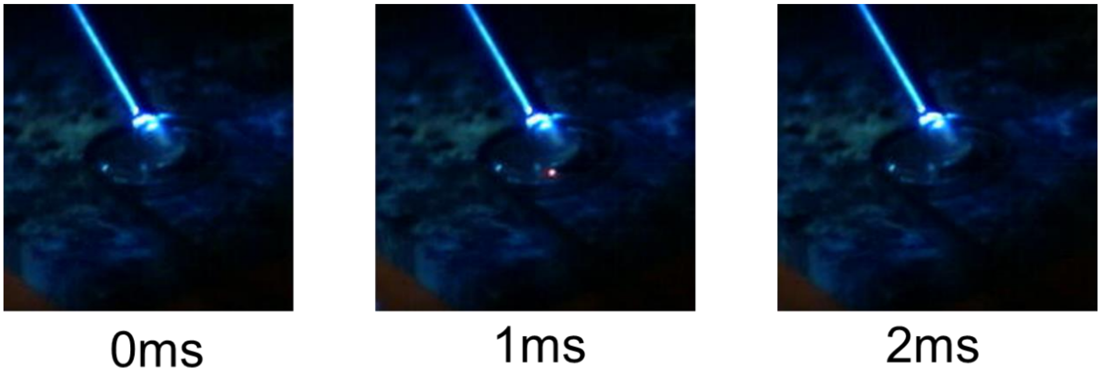

Supplementary Fig. 9. Lithiothermal reaction sparks were shot by ultra-speed camera (1000 pictures per second). A series of three pictures with same interval of 1ms demonstrate that the lithiothermal reaction can be accomplished in 1 ms.

Note: The forming of a carbon nanostructure such as fullerene during condensation is in tens picoseconds time scale (*3*).

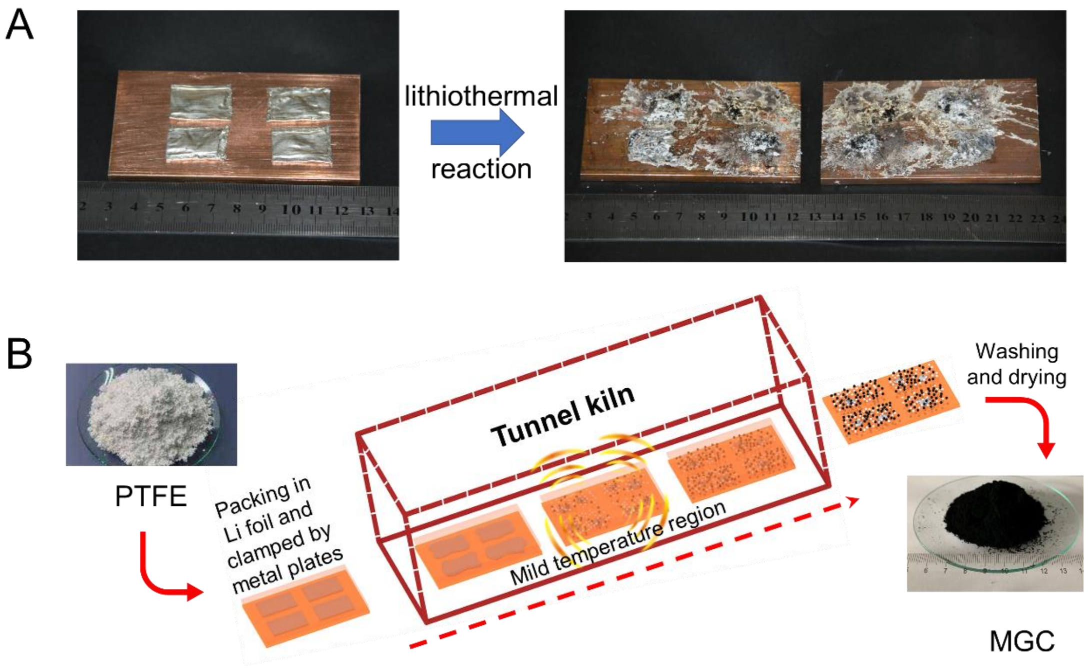

Supplementary Fig. 10. Large scale production of MGC by lithiothermal method. (A) Optical image of batch of 4 Li wrapper/PTFE filling envelops for MGC production. **(B)** Schematic of large-scale production of MGC by tunnel kiln. The image consists of approximately 3 g of sample.

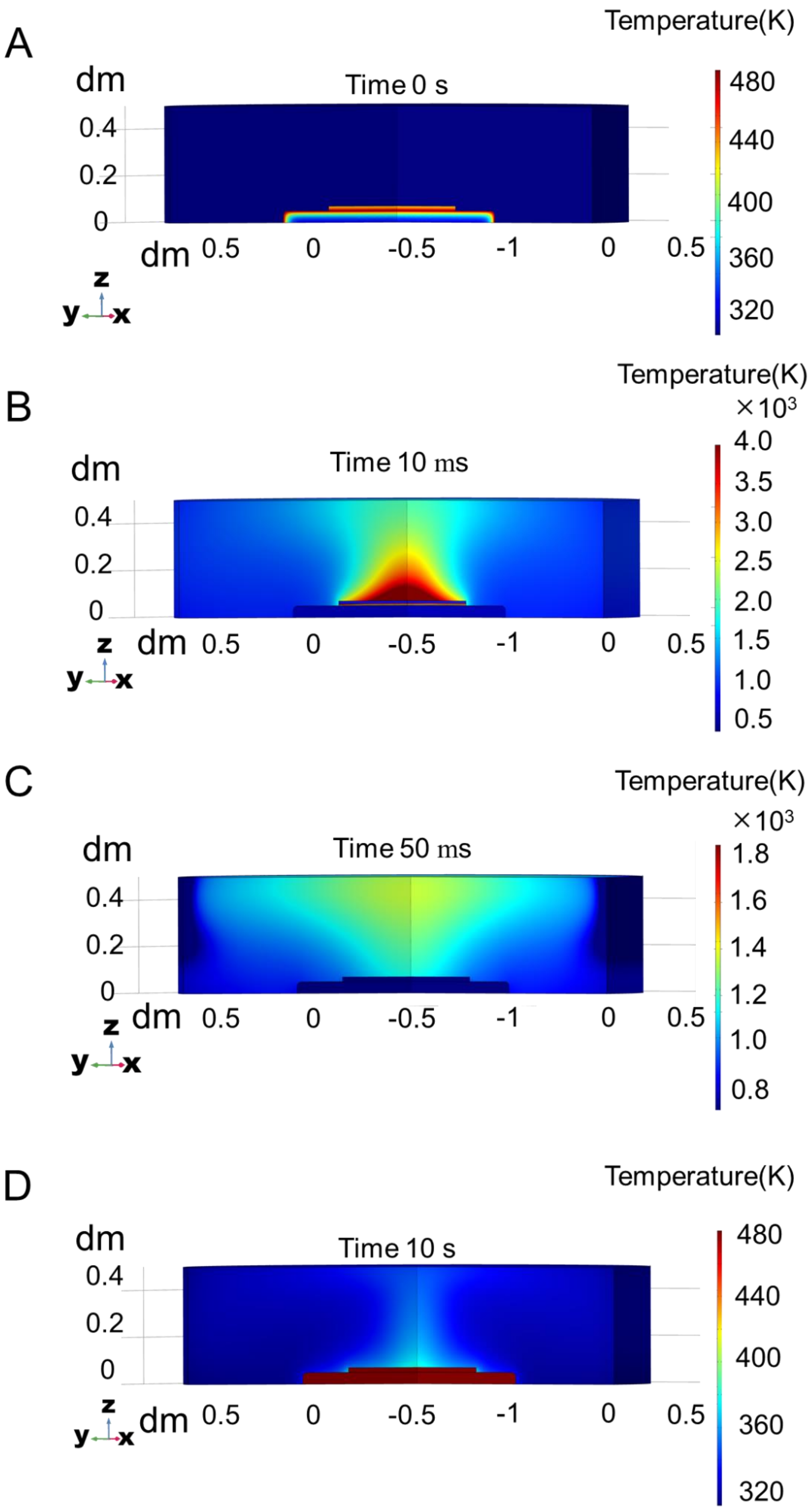

Supplementary Fig. 11. Numerical simulation results for temperature field of Lithiothermal reaction at different time. The simulation results displayed an obvious high temperature reaction zone (>3000 K) can be realized in 10ms, and the reaction is finished rapidly.

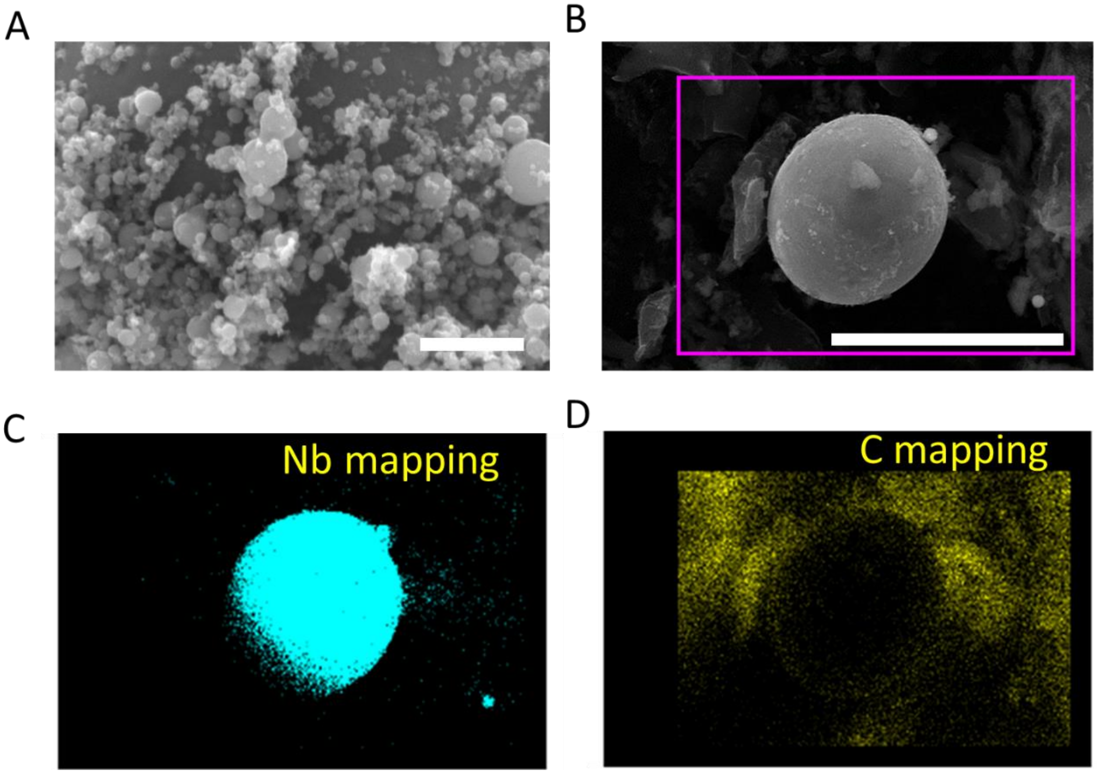

Supplementary Fig. 12. SEM and related niobium powder and carbon EDS images for the product after lithiothermal reaction(B-D). To further investigate the reaction temperature of lithiothermal reaction, niobium with melting point >2700 K was added in the system. Before reaction, the niobium shows particle size of 40-60 nm (A), while after the reaction, niobium particle with size larger than 8 μm appears (B). This phenomenon indicates the actual lithiothermal reaction temperature is at least higher than 2700 K, which can act as complementary proof to theoretical calculations. Scale bar: 500 nm (A) and 10 μm (B).

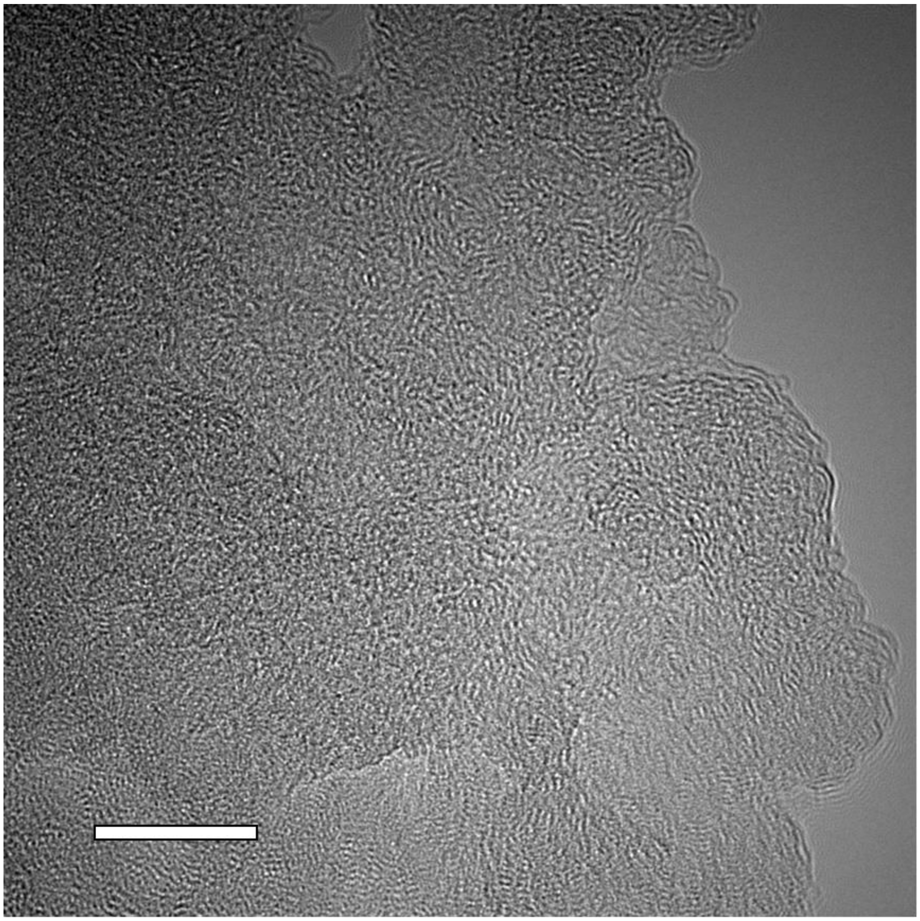

Supplementary Fig. 13. High-resolution TEM of MGC-5%. Scale bar: 10 nm.

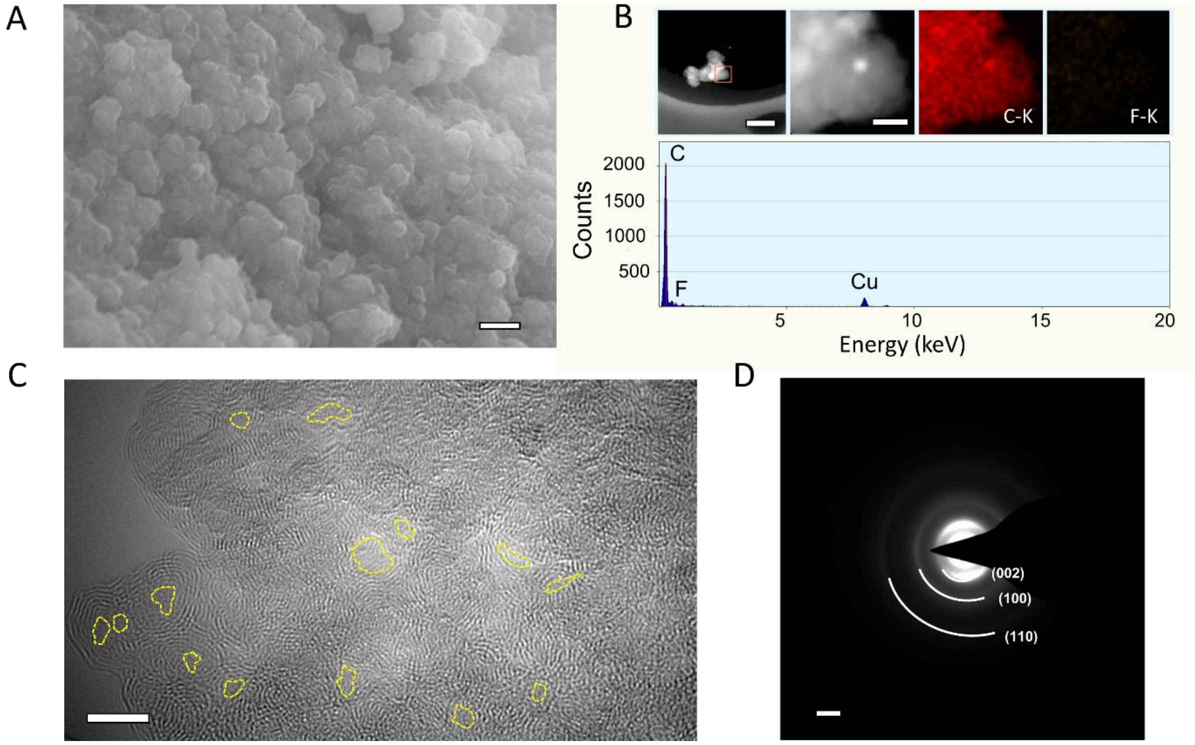

Supplementary Fig. 14. Characterization of porous graphitized carbon (MGC). (A) SEM image indicate the diameter of MGC agglomerates is ~100 nm. **(B)** EDS mapping of an MGC particle, demonstrating that the product is pure carbon product. **(C, D)** TEM image and corresponding selected-area electron diffraction patterns of MGC. Yellow dash lines indicate the interspace (~ 3 nm) among carbon onion Nanospheres. Scale bar: 200 nm (A), 500 nm and 100nm(enlarged view) (B) and 5 nm (C) and 2 1/nm (D).

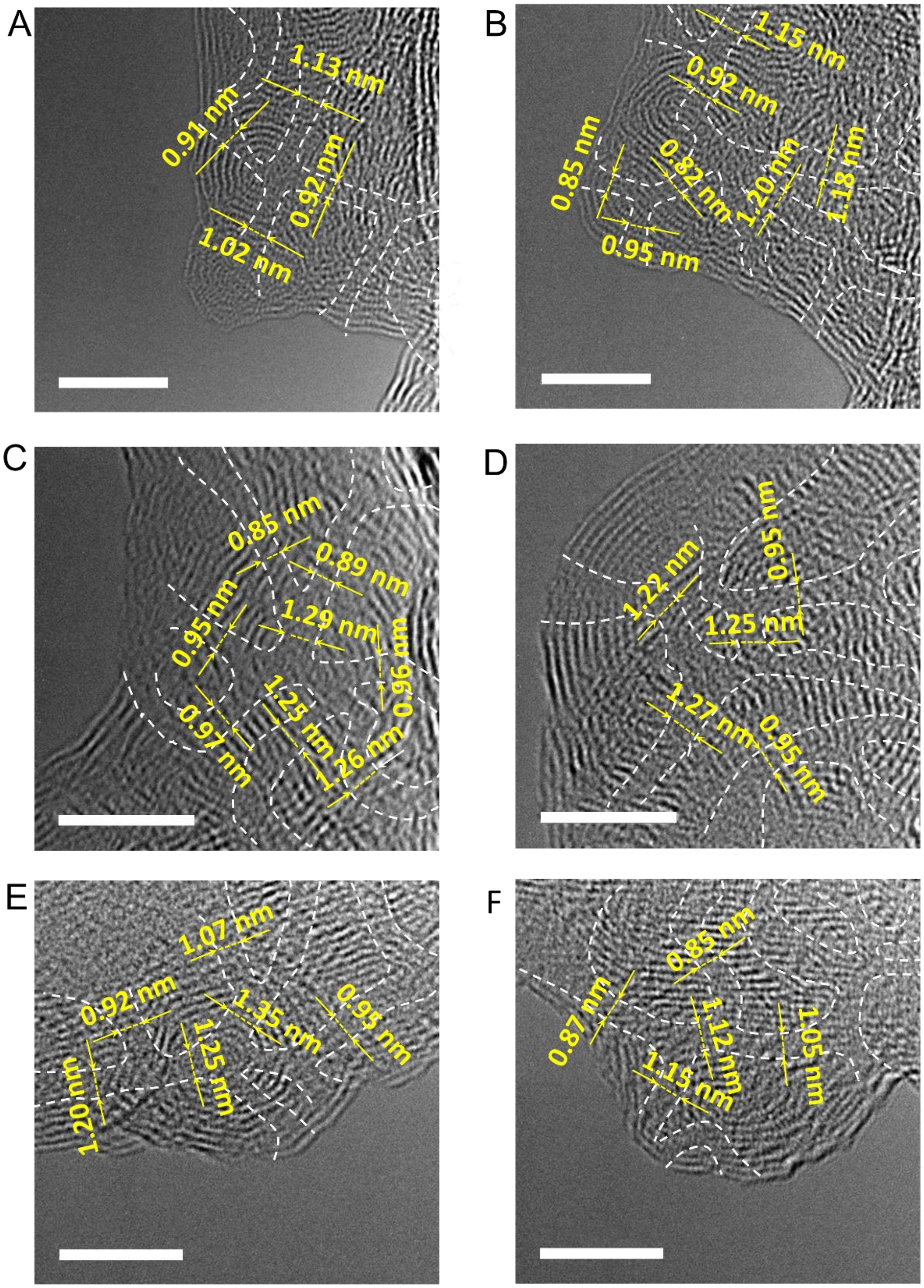

Supplementary Fig. 15. (A-F) Spherical aberration-corrected HRTEM images selected from different sites in MGC. Scale bar: 5nm (A-F). White dash lines indicate the cross-coupled open channel, which serves as ion-highways for fast ionic transport without block and dead ends.

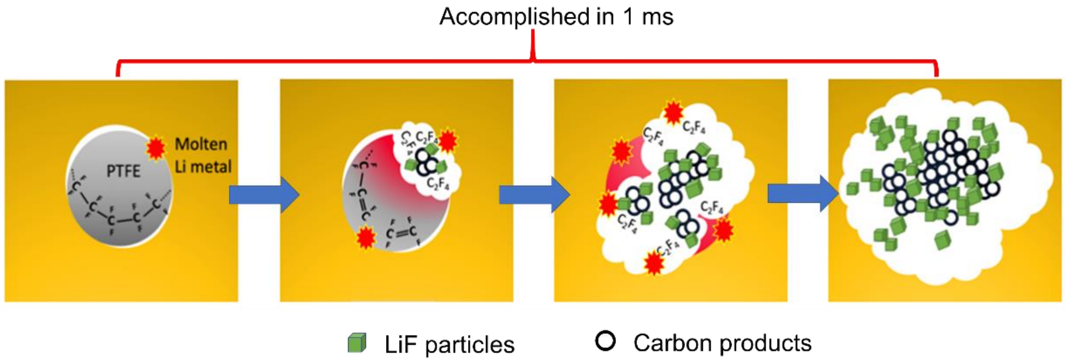

Supplementary Fig. 16. Reaction mechanism schematic between PTFE particle and molten Li metal. Once the reaction is triggered, large amount of gaseous CF_x (mainly C_2F_4) generates, providing large reaction interface with Li metal, which is favorable for the self-propagation in l ms.

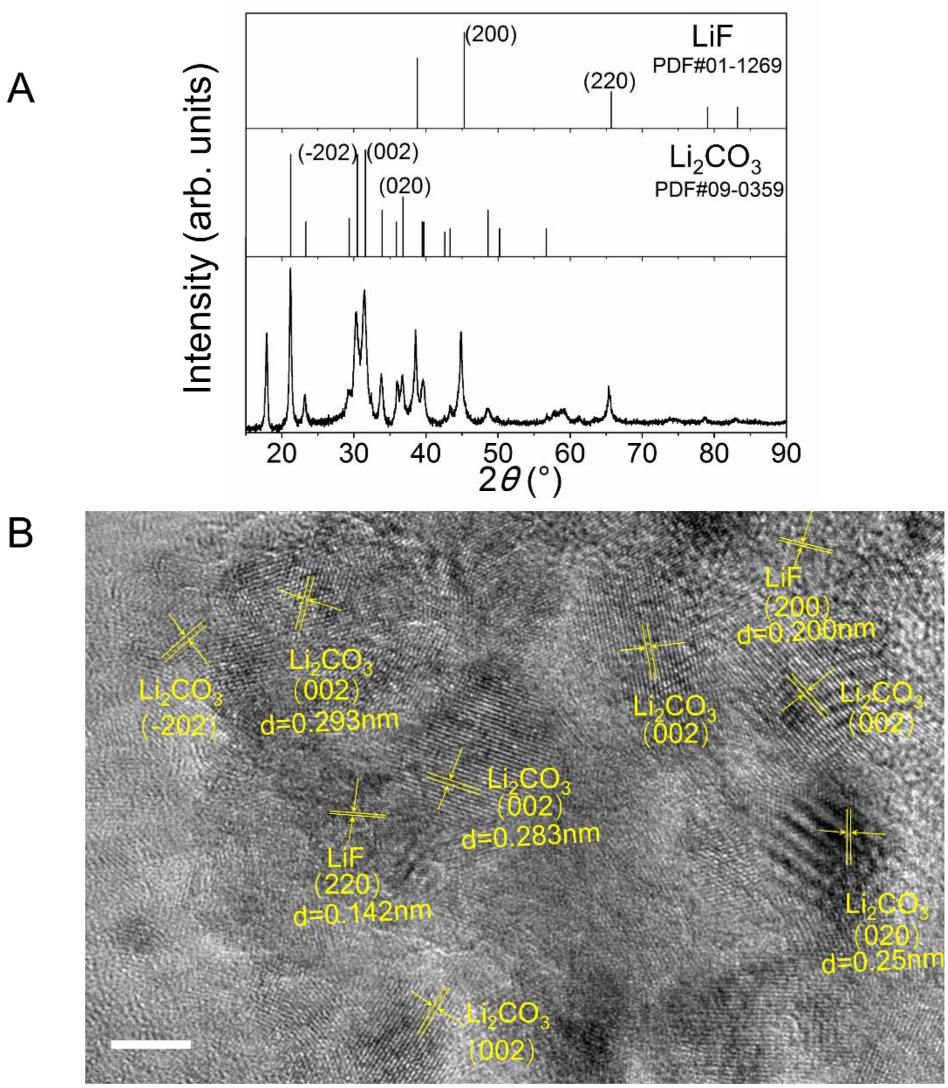

Supplementary Fig. 17. Characterization of lithiothermal reaction products in air. (A) XRD pattern indicates the existence of LiF and Li_2CO_3. **(B)** TEM image of the product. Tremendous LiF and Li_2CO_3 nanoparticles are observed. Scale bar: 5 nm.

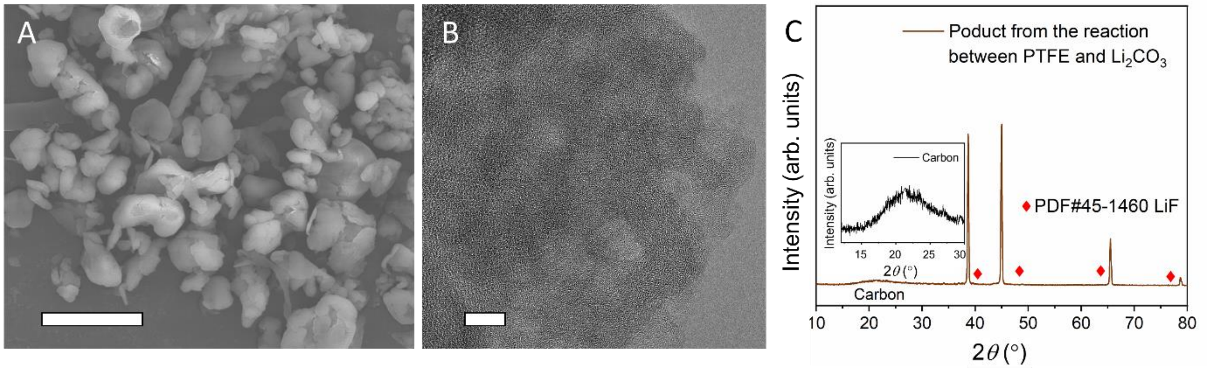

Supplementary Fig. 18. (A) SEM image of PTFE precursor. (B) TEM image and (C) XRD pattern of the product prepared from the reaction between PTFE precursor and Li_2CO_3. Scale bar: 5 μm (A) and 10 nm (B). Li_2CO_3 and PTFE were mechanical mixed at a stoichiometric ratio and pyrolyzed at 400 °C for 1 h under Ar atmosphere. It can be confirmed that LiF and amorphous carbon were formed through XRD. However, TEM image for the product shows only carbon material without LiF, which suggests that the LiF formed in the reaction was buried in the carbon material.

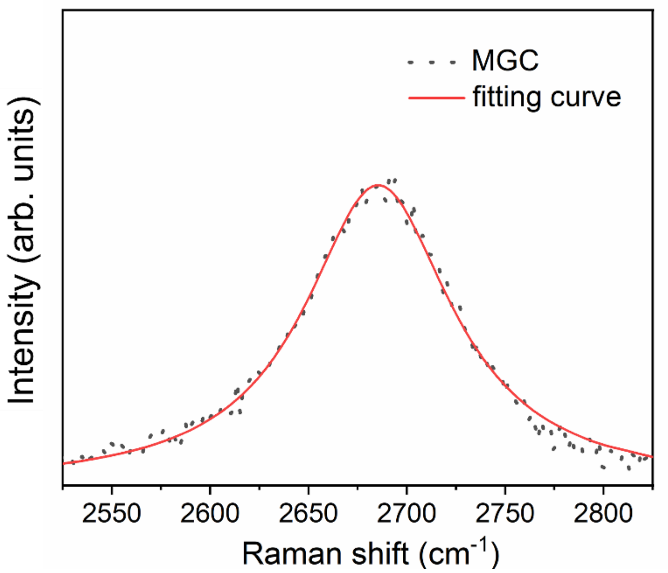

Supplementary Fig. 19. Raman spectrum of MGC sample. Fitted Raman spectra profile consists of a single Lorentzian component.

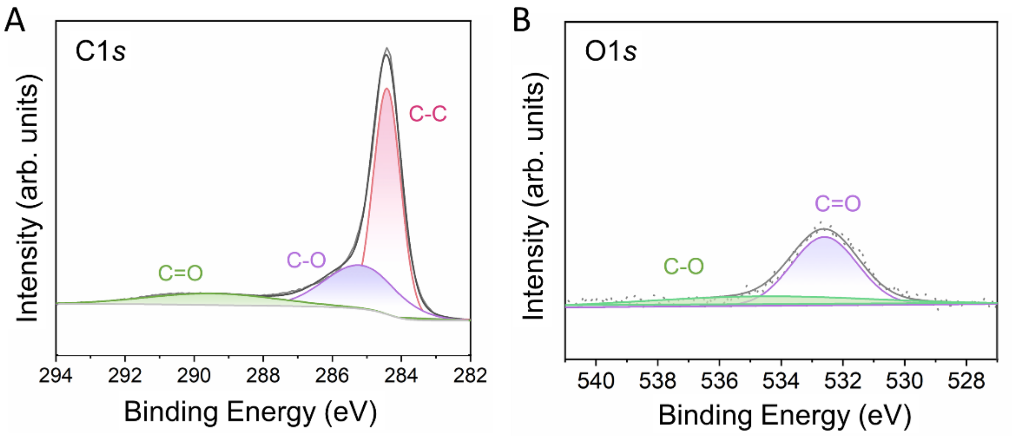

Supplementary Fig. 20. XPS (A) C1*s* and (B) O1*s* spectra of MGC.

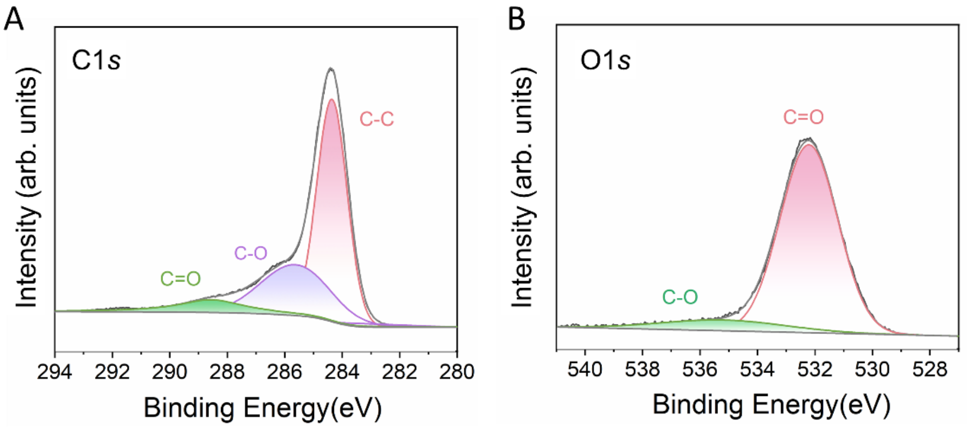

Supplementary Fig. 21. XPS (A) C1*s* and (B) O1*s* spectra of OMGC prepared in air.

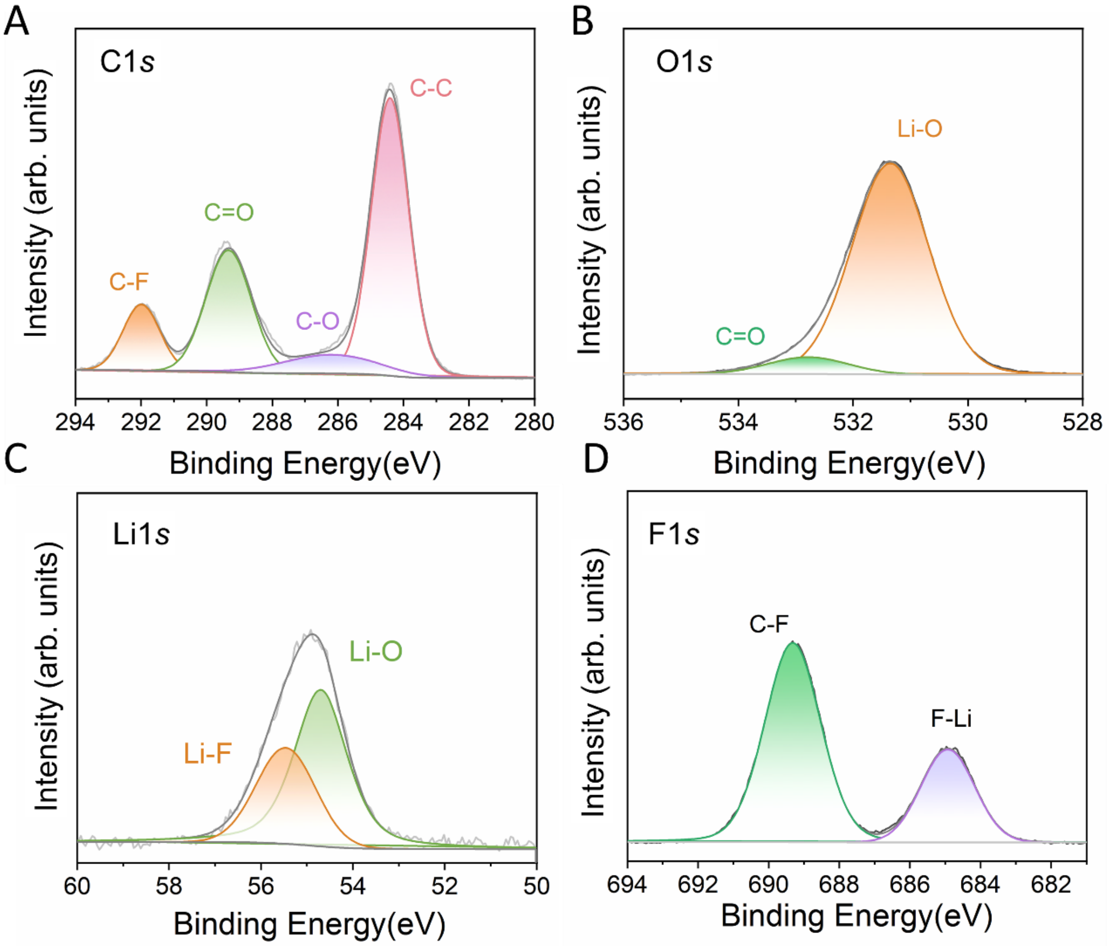

Supplementary Fig. 22. XPS (A) C1*s*, (B) O1*s*, (C) Li1*s* and (D) F1*s* spectra of lithiothermal reaction product in Ar. This sample is a lithiothermal reaction product without acid rinsing, which contains a large amount of lithium fluorine and some PTFE.

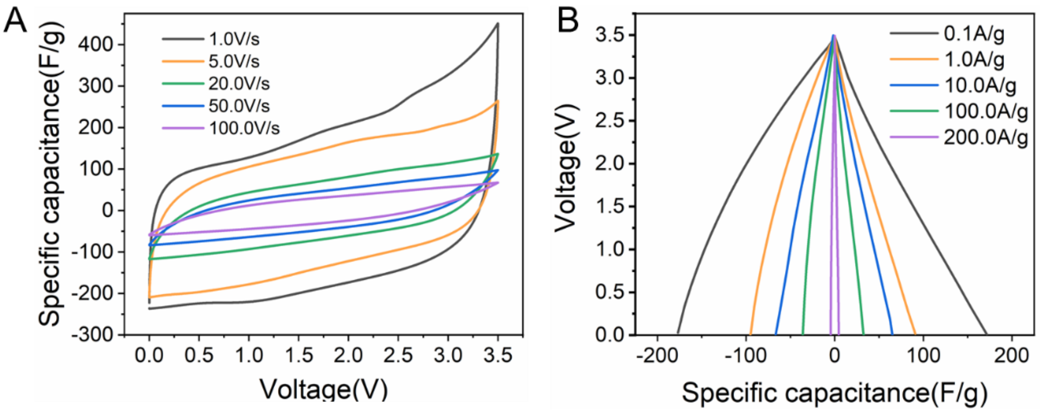

Supplementary Fig. 23. Supercapacitor performance of OMGC in $EMIMBF_4$ electrolyte. (A) CV curves from 1 to 100 V/s, which can sustain very high scan rates. **(B)** Galvanostatic charge/discharge curves at 0.1 to 200 A/g**.**

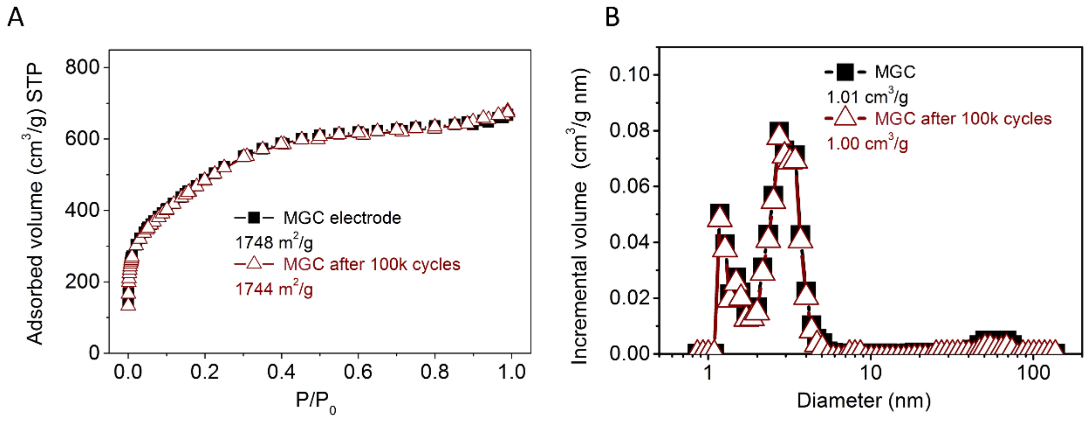

Supplementary Fig. 24. (A) N_2 adsorption/desorption isotherms and (B) pore size distribution of MGC electrode before and after 100,000 cycles.

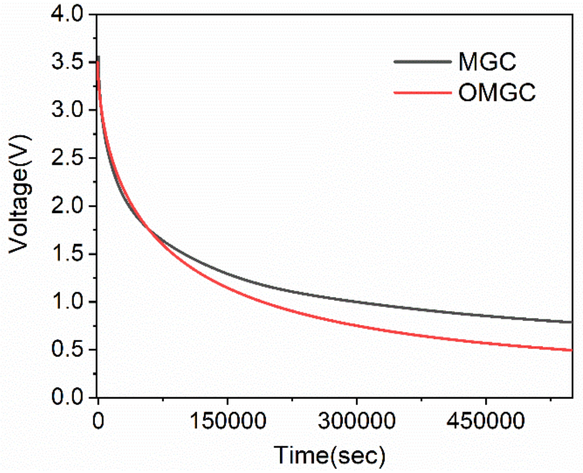

Supplementary Fig. 25. Self-discharge curves of MGC and OMGC at the charge current density of 1A/g.

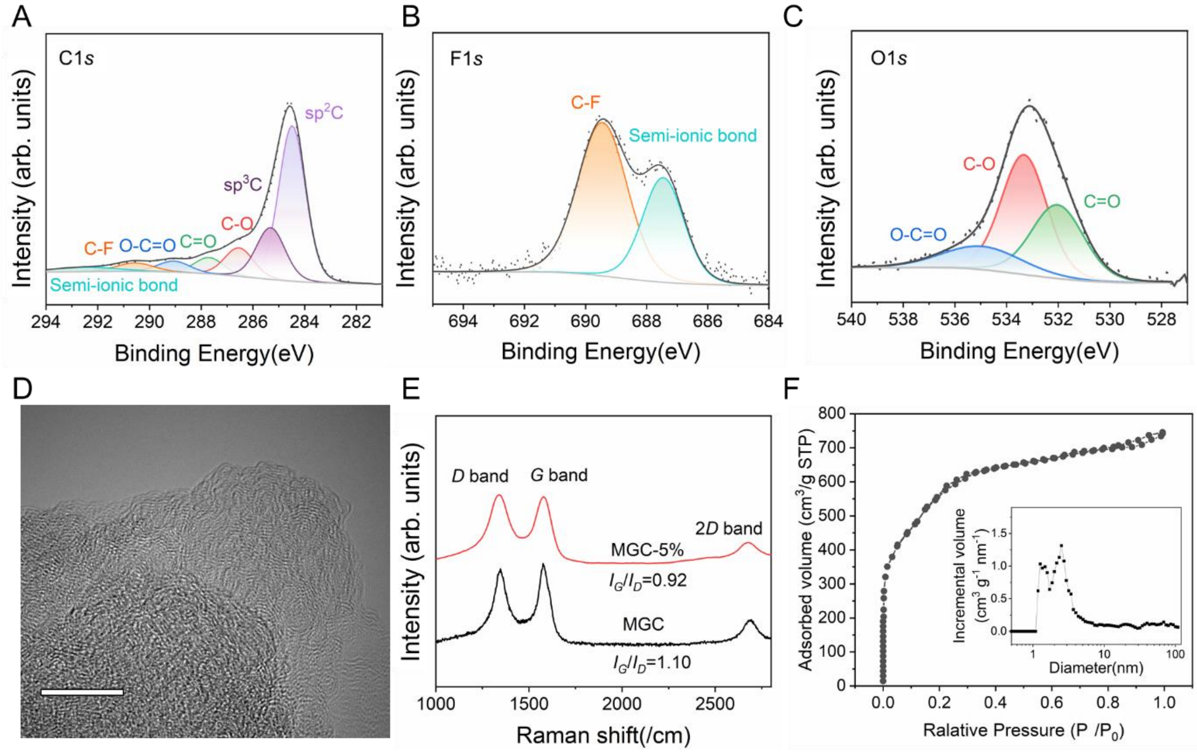

Supplementary Fig. 26. Characterization of MGC-5%. XPS **(A)** C1*s*, **(B)** F1*s* and **(C)** O1*s* spectra of MGC-5%, **(D)** High-resolution TEM, **(E)** Raman spectra and **(F)** N_2 adsorption/desorption isotherms and pore size distribution (inset). Scale bar: 10 nm (D).

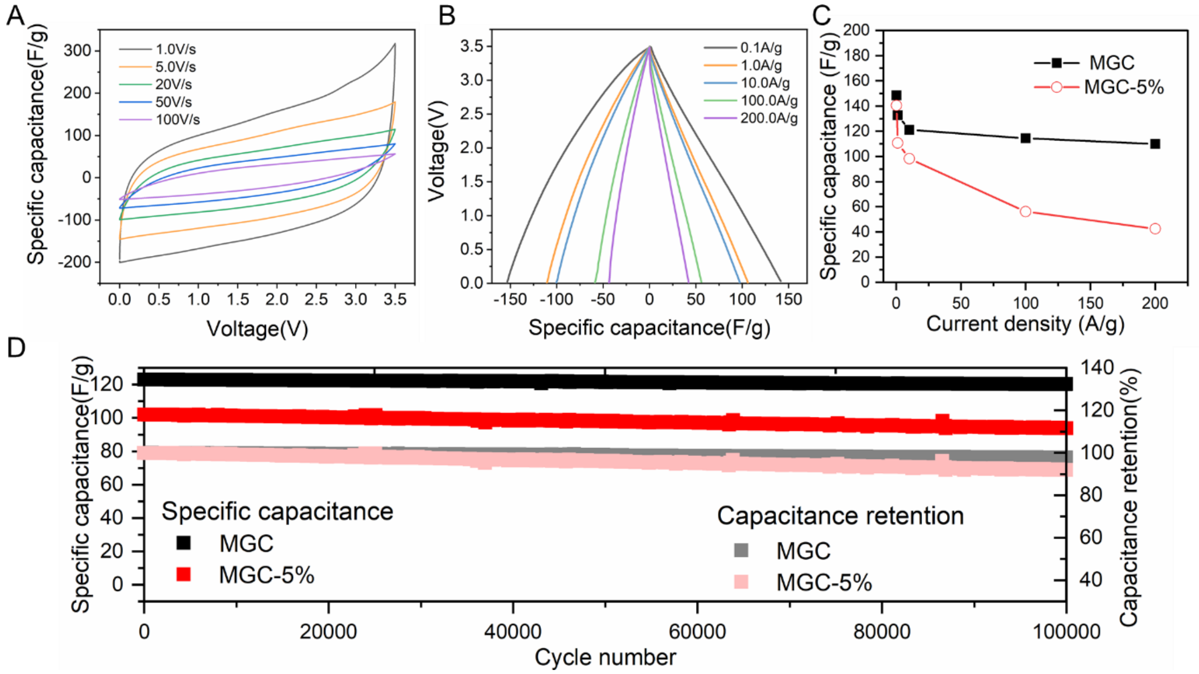

Supplementary Fig. 27. Supercapacitor performance of MGC-5% in $EMIMBF_4$ electrolyte. (A) CV curves from 1 to 100 V/s of MGC-5%, which can sustain very high scan rates. **(B)** Galvanostatic charge/discharge curves at 0.1 to 200 A/g for MGC-5%. **(C)** Variation of specific capacitance with charge/discharge current density of MGC and MGC-5%. **(D)** Capacitance retention for MGC-5% capacitors cycled at 10 A/g to 3.5V.

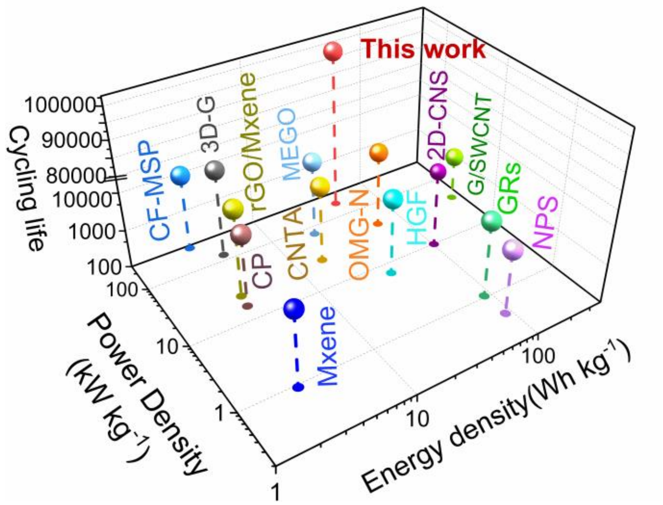

Supplementary Fig. 28. Comparative electrochemical performance of MGC sample and the best rate performance reported recently. CF-MSP: porous carbon foam electrode with multiscale pore network (3 M KOH) (*4*), 2D-CNS: 2D hierarchical porous carbon nanosheets ($EMIIMBF_4$) (*5*), CNTA: carbon nanotube arrays (1 M H_2SO_4) (*6*), MXene: two-dimensional titanium carbide (1 M H_2SO_4) (*7*); G/SWCNT: graphene/ SWCNT ($EMIMBF_4$) (*8*); 3D-G: 3D few-layer graphene ($EMIMBF_4$) (9), HGF: holey graphene framework ($EMIMBF_4$/AN) (*10*), CP: conducting polymer (PVA/H_3PO_4 gel) (*11*), rGO/Mxene: reduced graphene oxide/MXene (1 M H_2SO_4) (*12*), OMG-N: N-doped ordered porous carbon (0.5 M H_2SO_4) (*13*), MEGO: microwave exfoliated graphite oxide ($BMIMBF_4$/AN) (*14*), NPS: 2D nanoporous carbon sheets (1 M H_2SO_4/0.05 M KI) (*15*), GRs: graphene ribbon (0.1 M H_2SO_4) (*16*).

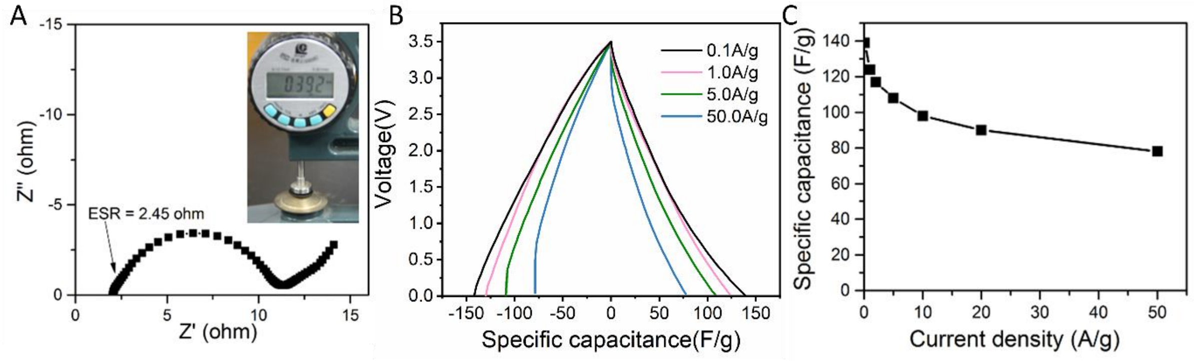

Supplementary Fig. 29. Supercapacitor performance of MGC with a high mass loading of 8.5 mg/cm^2 in EMIMBF$_4$ electrolyte. (A) Nyquist plots for MGC capacitors taken at 3.5 V. The insets show the thickness of MGC electrode. MGC electrode shows low ESR values (2.46 ohm). **(B)** Galvanostatic charge/discharge curves at 0.1 to 50 A g^{-1} for MGC. Internal resistance, especially at large discharge current density (50 A/g), is obvious. **(C)** Variation of specific capacitance with charge/discharge current density of MGC. Sample MGC gives the highest specific capacitance of 140 F/g at 0.1 A/g, and a specific capacitance of 81 F/g even at a high current density of 50 A/g, with a satisfactory capacitance retention of 57.9%.

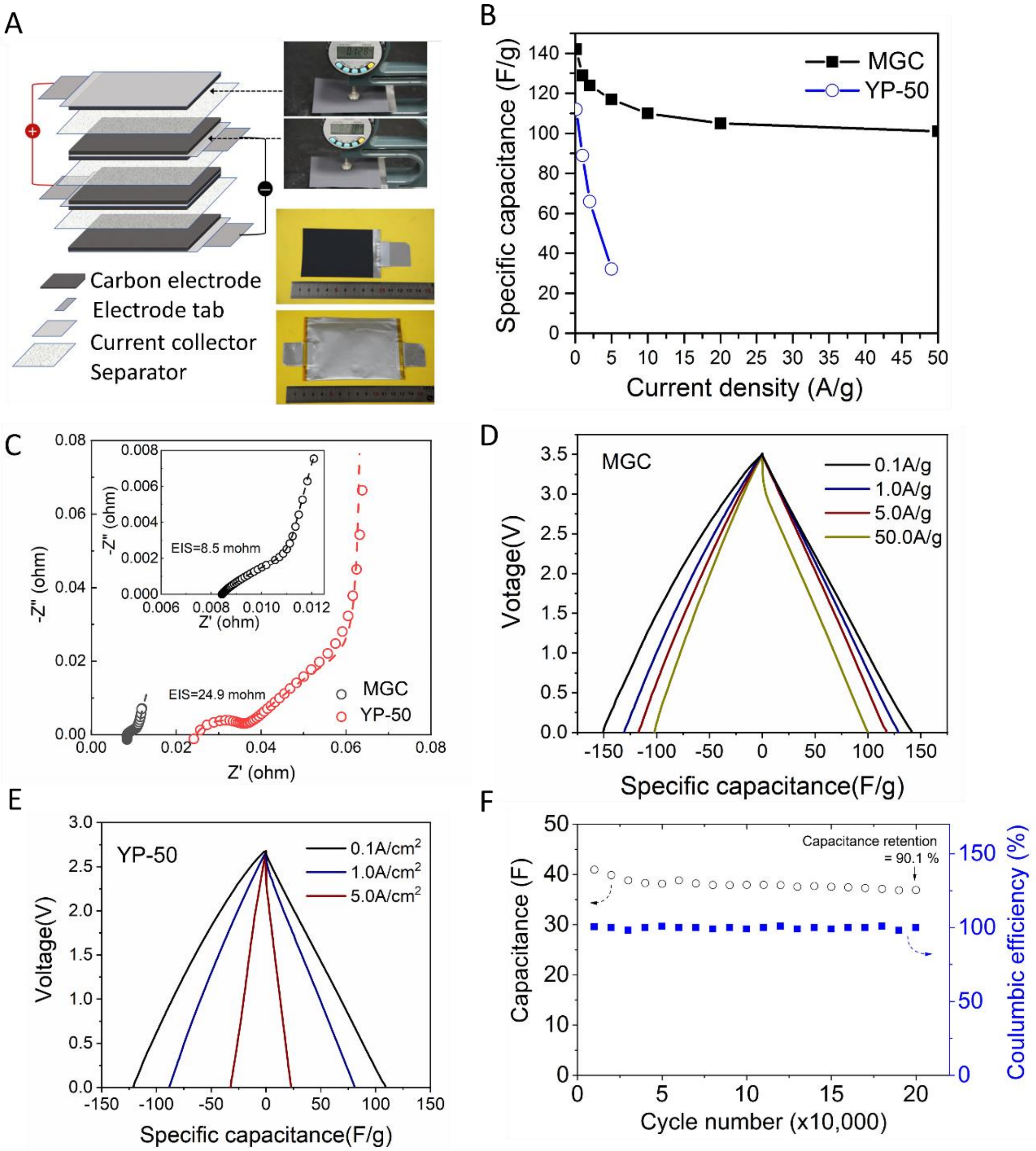

Supplementary Fig. 30. An electrochemical performance comparison for soft-package supercapacitor based on MGC and YP-50 in $EMIMBF_4$. Their corresponding loading density is 0.25 and 0.29 g cm^{-3}. **(A)** The digital images of soft-package device with internal parallel structure. **(B)** Variation of specific capacitance with charge/discharge current density. **(C)** Nyquist plots for MGC and YP-50 capacitors taken at 3.5 V and 2.75V, respectively. Galvanostatic charge/discharge curves at different current densities of **(D)** MGC and **(E)** YP-50. **(F)** Cyclic stability of MGC after 200,000 cycles at 20.0 A g^{-1} with a voltage window of 3.5 V.

The substantially better high-power performance of MGC supercapacitor is consistent with the its low ESR of 8.5 mohm, much lower than that of YP-50 (24.9 mohm) (Supplementary Fig. 30c), which is consistent with its minimum voltage drop in GCD studies (Supplementary Fig. 30d, e),

further manifesting its ultrahigh ion delivery capability. Furthermore, MGC capacitor presents impressive capacitance retention (over 90 %) at current of 20.0 A/g after 200,000 continuous cycles (Supplementary Fig. 30f), reflecting the stability and universality of the internal parallel architecture.

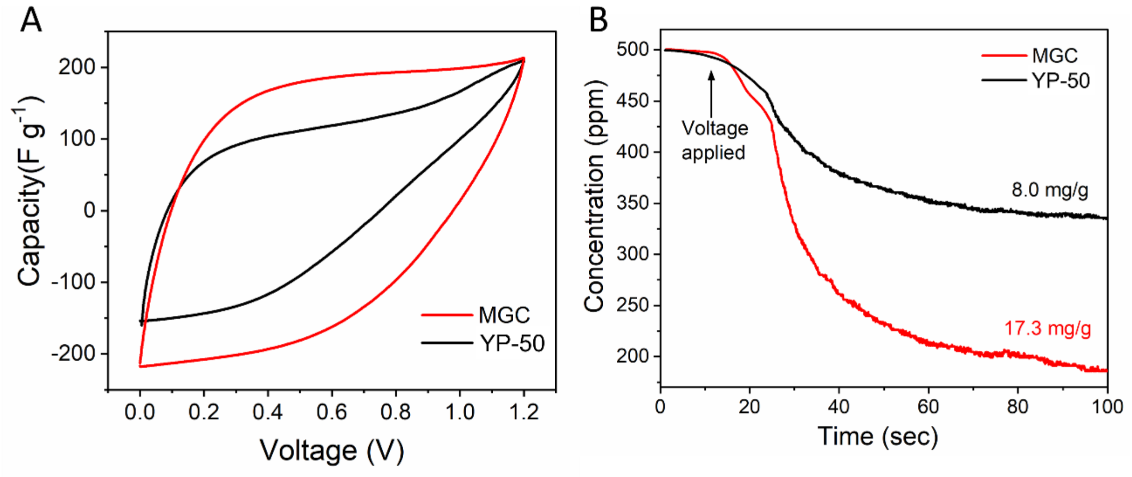

Supplementary Fig. 31. Capacitive Deionization performance. (A) CV curves of MGC and YP-50 (activated carbon) measured at a scan rate of 1 mVs^{-1}. Obviously, MGC showed a larger integral area than that of YP-50 under the same test conditions that revealed the higher specific capacitance of MGC. **(B)** Concentration changes of the effluent with time for the MGC and YP-50 based cells treated by applying 1.0 V when desalinating 500 ppm NaCl aqueous solution. The ion removal capacity during the 100 s ion capturing process was 17.3 mg/g, more than two times than that of the YP-50 electrode.

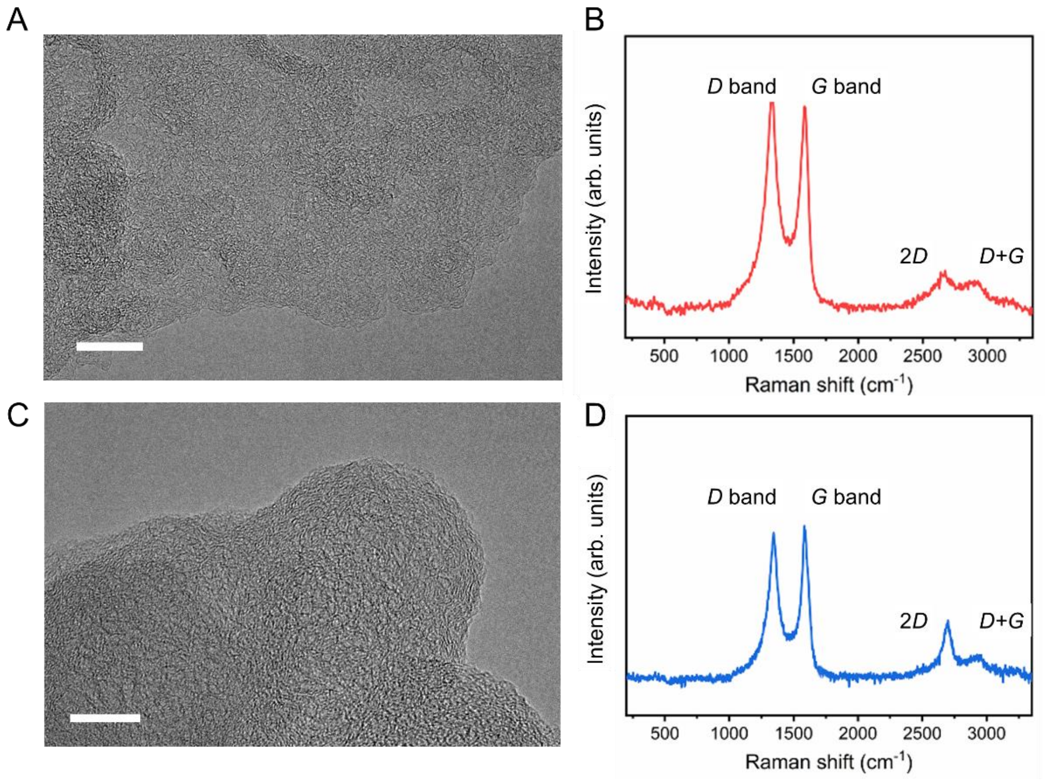

Supplementary Fig. 32. Structure of porous carbon material prepared from Na/K and PTFE. **(A)** High-resolution TEM and **(B)** Raman spectra of carbon product from the reaction between PTFE and Na. **(C)** High-resolution TEM and **(D)** Raman spectra of carbon product from the reaction between PTFE and K. The preparation process is similar with that of PTFE and Li. The products from the reaction between PTFE and Na/K were also porous and highly graphitized. Their difference is worth pursuing in future. Scale bar: 10 nm (A) and (C).

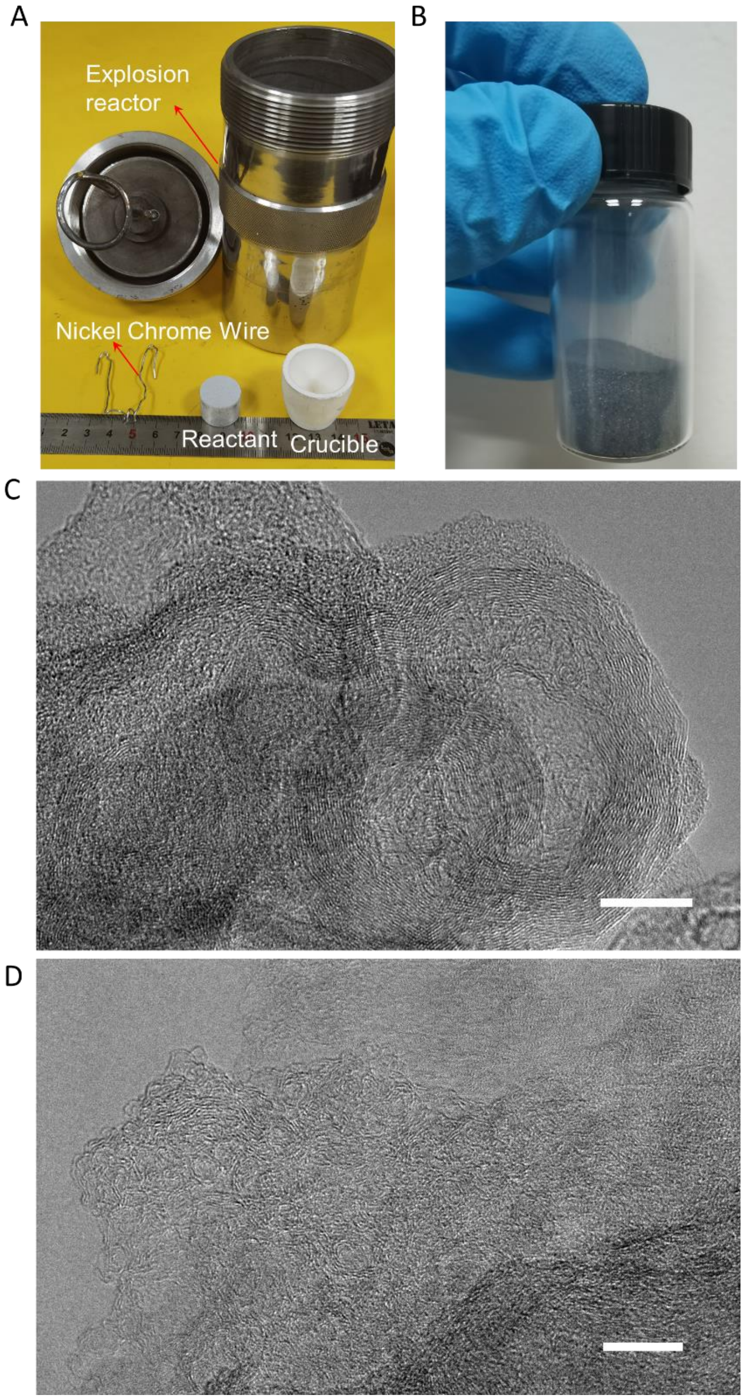

Supplementary Fig. 33. carbon materials prepared from Mg (or Al) and PTFE. Mg (or Al) and PTFE powder were compacted into reactant pillar under a pressure of 2 MPa, which was put into a crucible in the reactor **(A)** (*17*). Then a 10 A current was exerted through the nickel chrome wire for 5 seconds to produce enough heat for the initiation of the reaction. After the reaction, the collected powder was rinsed in 1 M HNO_3 and deionized water to obtain the carbon product **(B)**. TEM images of the carbon product from the reaction between PTFE and Mg **(C)**, Al **(D)**. Scale bar: 10 nm (C) and (D).

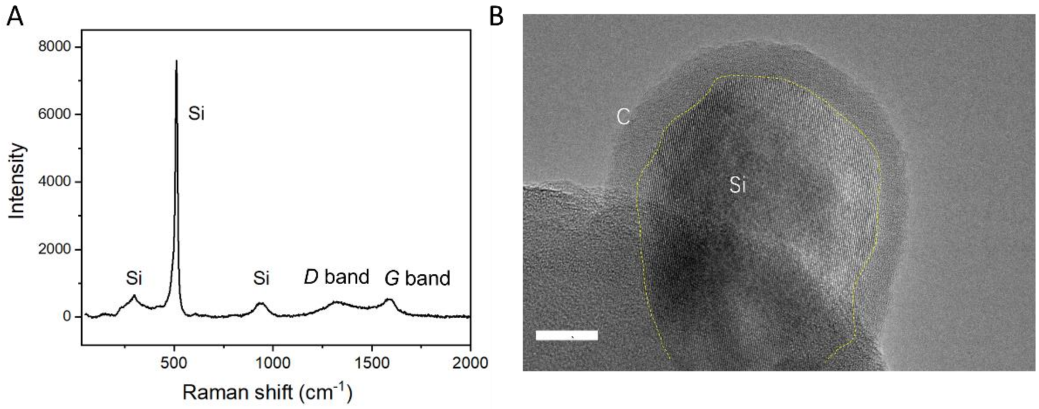

Supplementary Fig. 34. Structure of Si/C material from Si and PTFE. (A) Raman spectra and **(B)** High-resolution TEM of Si/C material. Scale bar: 5nm. The Si/C material was prepared in a similar method with Supplementary Fig. 33: Si and PTFE powder were compacted into reactant pillar under a pressure of 2 MPa, which was put into a crucible in the reactor of Supplementary Fig. 33A. Then a 10 A current was exerted through the nickel chrome wire for 5 seconds to produce enough heat for the initiation of the reaction and Si/C material was obtained.

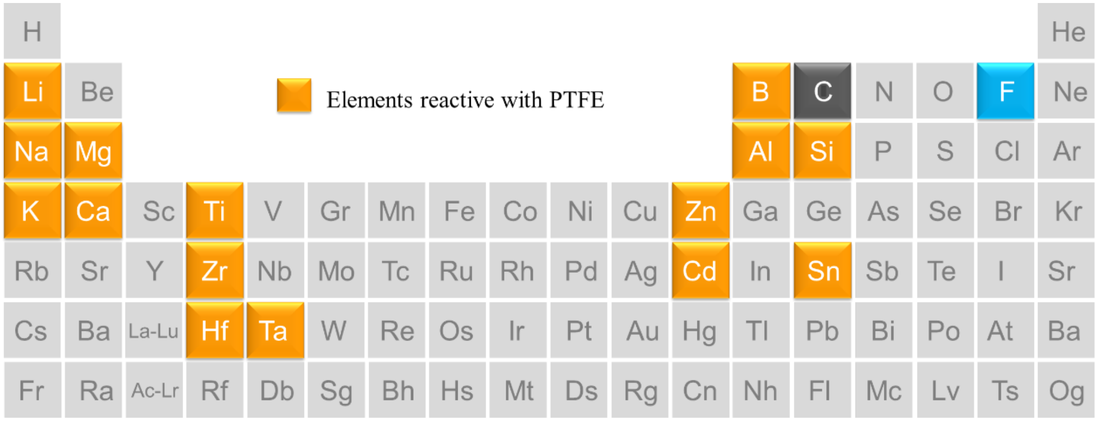

Supplementary Fig. 35. Key elements reactive with PTFE *(18)*. The lithiothermal strategy has been demonstrated to be applicable to Li, Na, K, Mg, Al, Si successfully to produce highly graphitized porous carbon materials.

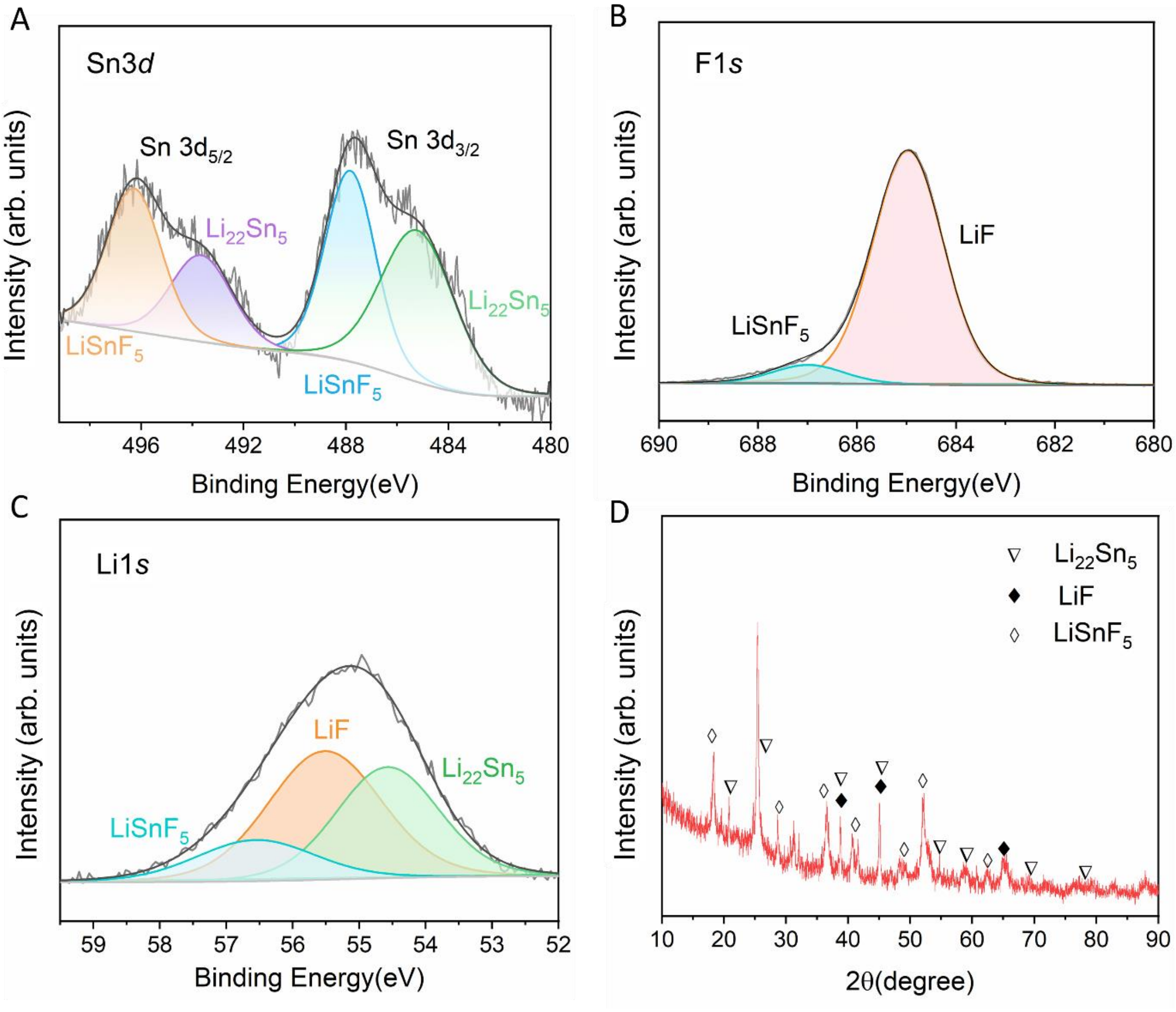

Supplementary Fig. 36. High-resolution XPS spectra of (a) Sn 3*d*, (b) F 1*s* and (c) Li 1*s* for the products from the reaction between Li metal and SnF_4. (d) XRD pattern XPS for the products from the reaction between Li metal and SnF_4. Tests were conducted to reveal the component information of the $Li_{22}Sn_5$/LiF/$LiSnF_5$ composite. Two main peaks at 485.2 eV and 493.6 eV could be assigned to Sn $3d_{3/2}$ and Sn $3d_{5/2}$ of $Li_{22}Sn_5$, and the other two peaks at 487.8 eV and 496.2 eV indicate Sn $3d_{3/2}$ and Sn $3d_{5/2}$ of $LiSnF_5$ (Supplementary Fig. 36a). The F 1s peak at 685.1 eV (Supplementary Fig. 36b) and the deconvoluted Li 1s peak at 55.6 eV (Supplementary Fig. 36c) indicated the existence of LiF. The remained two peaks at 54.5 eV and 56.7 eV in Li 1s spectra corresponded to the $Li_{22}Sn_5$ and $LiSnF_5$, respectively. The reaction was conducted as follows: SnF_4 was enveloped into Li bag, and clamp it using two Cu plates with 1 MPa. Then Cu plates were heated to 200 °C followed by repeated friction, the reaction was immediately initiated and completed within millisecond

Supplementary Table 1. Pore structure parameters from the carbon prepared by 1 μm-PTFE precursor.

Sample	S_{BET} ($m^2\ g^{-1}$) [a]	Pore volume ($10^{-3} cm^3\ g^{-1}$)		
		V_{total}[b]	V_{micro}[c]	V_{meso}[d]
1	2081	1.24	0.32	0.60

[a] S_{BET}: Specific surface area. [b] V_{total}: Total pore volume measured at $P/P_0 = 0.99$. [c, d] V_{micro}, V_{meso}: Micropore volume and mesopore volume determined by DFT model.

Supplementary Table 2. Element content (%) of various materials*

Sample	C	O	F	Li
MGC	98.8	1.2	/	/
MGC-products	31.6	27.2	15.4	25.8
OMGC	82.5	17.5	/	/

*Atomic content (%)

Supplementary Table 3. Performance of selected porous carbon materials for ECs

Electrode materials	Electrolyte	Capacitance (rate)	Energy density	**Power density**	Capacitance retention (no. of cycles)	Ref
Mxene	H_2SO_4	245 F g^{-1} (2 mV s^{-1})	2.6 Wh kg^{-1}	0.93 kW kg^{-1}	100% (10000)	(*7*)
Graphene/ SWCNT (G/SWCNT)	[EMIM][BF_4]	199 F g^{-1} (0.5 A g^{-1})	88 Wh kg^{-1}	1.05 kW kg^{-1}	98% (10000)	(*8*)
Activated carbon/poly(3-methylthiophene) e) (a-PMT)	$PYR_{14}TFSI$	428 F g^{-1} (10 mA cm^{-2})	14 Wh kg^{-1}	1.9 kW kg^{-1}	90% (16000)	(*19*)
MoS_2-C	NaCl/H_2O	416 F g^{-1} (1 A g^{-1})	13 Wh kg^{-1}	3.2 kW kg^{-1}	100% (50000)	(*20*)
Holey graphene framework (HGF)	[EMIM][BF_4]/AN	298 F g^{-1} (1 A g^{-1})	35 Wh kg^{-1}	7 kW kg^{-1}	91% (10000)	(*10*)
Conducting Polymer (CP)	PVA/H_3PO_4 gel	120 F g^{-1} (0.4 A g^{-1})	3.2 Wh kg^{-1}	16.2 kW kg^{-1}	80% (8000)	(*11*)
rGO/Mxene	H_2SO_4	85 F g^{-1} (2 mV s^{-1})	3.3 Wh kg^{-1}	24 kW kg^{-1}	100% (20000)	(*12*)
N-doped ordered mesoporous carbon (OMG-N)	H_2SO_4	850 F g^{-1} (1 A g^{-1})	63Wh kg^{-1}	44 kW kg^{-1}	92% (10000)	(*13*)
Microwave exfoliated GO (MEGO)	[BMIM][BF_4]/AN	166 F g^{-1} (5.7 A g^{-1})	21 Wh kg^{-1}	75 kW kg^{-1}	97% (10000)	(*14*)
Graphene/ethyl cellulose (G/EC)	H_3PO_4/PVA gel	63.6 F g^{-1} (0.5 A cm^{-3})	2.2 mWh g^{-1}	239 kW kg^{-1}	95% (10000)	(*21*)
Carbide-derived carbon (CDC)	$TEABF_4$/AN	130 F g^{-1} (5 mA cm^{-2})	28 Wh kg^{-1}	0.84 kW kg^{-1}	N/A	(*22*)
Laser-scribed graphene (LSG)	[TEA][BF_4]/AN	4.82 mF cm^{-2} (1 A g^{-1})	0.7 mWh cm^{-3}	200 mW cm^{-3}	96.5% (10000)	(*23*)

Supplementary Table 3. (cont.) Performance of selected porous carbon materials for ECs

Electrode materials	Electrolyte	Capacitance (rate)	Energy density	**Power density**	Capacitance retention (no. of cycles)	Ref
Porous oxo-carbon	H_2SO_4	90 F g^{-1} (10 A g^{-1})	5 Wh kg^{-1}	2.5kW kg^{-1}	94.7% (10000)	(*24*)
Carbon Superstructures	H_2SO_4	468 F g^{-1} (1 A g^{-1})	12 Wh kg^{-1}	10 kW kg^{-1}	93.1 (1000000)	(*25*)
Ultrafine structure-activated porous carbon	$EMIMBF_4$	80 F g^{-1} (100A g^{-1})	71 Wh kg^{-1}	17 kW kg^{-1}	99% (4000)	(*26*)
**MGC**	**$EMIMBF_4$**	**110 F g^{-1} (200 A g^{-1})**	**47 Wh kg^{-1}**	**175 kW kg^{-1}**	**97.7% (100000)**	**This work**

Supplementary References

1. Q. Dong et al., Programmable heating and quenching for efficient thermochemical synthesis. *Nature* **605**, 470-476 (2022).
2. M. L. Oshea, C. Morterra, M. J. D. Low, Spectroscopic studies of carbons. XVII. Pyrolysis of polyvinylidene fluoride. *Mater. Chem. Physics* **26**, 193-205 (1990).
3. S. Irle, G. Zheng, Z. Wang, K. Morokuma, The C-60 formation puzzle "solved": QM/MD simulations reveal the shrinking hot giant road of the dynamic fullerene self-assembly mechanism. *J. Physical Chem. B* **110**, 14531-14545 (2006).
4. F. Zhang et al., Multiscale Pore Network Boosts Capacitance of Carbon Electrodes for Ultrafast Charging. *Nano Lett.* **17**, 3097-3104 (2017).
5. L. Yao et al., Scalable 2D Hierarchical Porous Carbon Nanosheets for Flexible Supercapacitors with Ultrahigh Energy Density. *Adv. Mater.* **30**, 1706054 (2018).
6. G. Xiong et al., Bioinspired leaves-on-branchlet hybrid carbon nanostructure for supercapacitors. *Nat. Commun.* **9**, 790 (2018).
7. M. Ghidiu, M. R. Lukatskaya, M.-Q. Zhao, Y. Gogotsi, M. W. Barsoum, Conductive two-dimensional titanium carbide 'clay' with high volumetric capacitance. *Nature* **516**, 78-U171 (2014).
8. X. Yang, C. Cheng, Y. Wang, L. Qiu, D. Li, Liquid-Mediated Dense Integration of Graphene Materials for Compact Capacitive Energy Storage. *Science* **341**, 534-537 (2013).
9. J. Zhao et al., Porous 3D Few-Layer Graphene-like Carbon for Ultrahigh-Power Supercapacitors with Well-Defined Structure-Performance Relationship. *Adv. Mater.* **29**, 1604569 (2017).
10. Y. Xu, Z. Lin, X. Zhong, X. Huang, N. O. Weiss, Y. Huang, X. Duan, Holey graphene frameworks for highly efficient capacitive energy storage. *Nat. Commun.* **5**, 4554 (2014).
11. Z. Li et al., Free-Standing Conducting Polymer Films for High-Performance Energy Devices. *Angew. Chem. Int. Ed.* **55**, 979-982 (2016).
12. J. Yan et al., Flexible MXene/Graphene Films for Ultrafast Supercapacitors with Outstanding Volumetric Capacitance. *Adv. Funct. Mater.* **27**, 1701264 (2017).
13. T. Lin et al., Nitrogen-doped mesoporous carbon of extraordinary capacitance for electrochemical energy storage. *Science* **350**, 1508-1513 (2015).
14. Y. Zhu et al., Carbon-Based Supercapacitors Produced by Activation of Graphene. *Science* **332**, 1537-1541 (2011).
15. K. Jayaramulu et al., Ultrathin Hierarchical Porous Carbon Nanosheets for High-Performance Supercapacitors and Redox Electrolyte Energy Storage. *Adv. Mater.* **30**, 1705789 (2018).
16. A. K. Farquhar, M. Supur, S. R. Smith, C. Van Dyck, R. L. McCreery, Hybrid Graphene Ribbon/Carbon Electrodes for High-Performance Energy Storage. *Adv. Energy Mater.* **8**, 1802439 (2018).
17. P. Ghildiyal et al., Silicon Nanoparticles for the Reactivity and Energetic Density Enhancement of Energetic-Biocidal Mesoparticle Composites. *Acs Appl. Mater. Interfaces* **13**, 458-467 (2021).
18. E.-C. Koch, Metal-fluorocarbon based energetic materials. *Wiley-VCH: Weinheim*, Germany (2012).
19. A. Balducci, W. Henderson, M. Mastragostino, S. Passerini, P. Simon, F. Soavi, Cycling stability of a hybrid activated carbon//poly(3-methylthiophene) supercapacitor with N-butyl-Nmethylpyrrolidinium bis(trifluoromethanesulfonyl)imide ionic liquid as electrolyte. *Electrochim. Acta* **50**, 2233-2237 (2005).
20. A. Gigot, M. Fontana, M. Serrapede, M. Castellino, S. Bianco, M. Armandi, B. Bonelli, C. Fabrizio Pirri, T. Elena, P. Rivolo, Mixed 1T-2H Phase MoS_2/Reduced Graphene Oxide as Active Electrode for Enhanced Supercapacitive Performance. *ACS Applied Mater. Interfaces* **8**, 32842 (2016).
21. L. Li, E. B. Secor, K.-S. Chen, J. Zhu, X. Liu, T. Z. Gao, J.-W. T. Seo, Y. Zhao, M. C. Hersam, High-

Performance Solid-State Supercapacitors and Microsupercapacitors Derived from Printable Graphene Inks. *Adv. Energy Mater.* **6**, 1600909 (2016).

22. J. Chmiola, G. Yushin, Y. Gogotsi, C. Portet, P. Simon, P. L Taberna, Anomalous Increase in Carbon Capacitance at Pore Sizes Less Than 1 Nanometer. *Science* **313**, 1760-1763 (2006).
23. M. El-Kady, V. Strong, S. Dubin, R. Kaner, Laser Scribing of High-Performance and Flexible Graphene-Based Electrochemical Capacitors. *Science* **335**, 1326-1330 (2012).
24. Li, J.; Kossmann, J.; Zeng, K.; Zhang, K.; Wang, B.; Weinberger, C.; Antonietti, M.; Odziomek, M.; López-Salas, N. When high-temperature cesium chemistry meets self-templating: Metal acetates as building blocks of unusual highly porous carbons. *Angew. Chem. Int. Ed.* **62**, e202217808 (2023).
25. Song, Z.; Miao, L.; Ruhlmann, L.; Lv, Y.; Zhu, D.; Li, L.; Gan, L.; Liu, M. Self-assembled carbon superstructures achieving ultra-stable and fast proton-coupled charge storage kinetics. *Adv. Mater.*, **33**, 2104148 (2021).
26. Zhang, J.; Luo, J.; Guo, Z.; Liu, Z.; Duan, C.; Dou, S.; Yuan, Q.; Liu, P.; Ji, K.; Zeng, C.; Xu, J.; Liu, W. D.; Chen, Y.; Hu, W. Ultrafast manufacturing of ultrafine structure to achieve an energy density of over 120 wh kg^{-1} in supercapacitors. *Adv.* Energy *Mater.* **13**, 2203061 (2022).
